# Targeted PMP22 TATA-box editing by CRISPR/Cas9 reduces demyelinating neuropathy of Charcot-Marie-Tooth disease type 1A in mice

**DOI:** 10.1093/nar/gkz1070

**Published:** 2019-11-12

**Authors:** Ji-Su Lee, Jae Y Lee, Dong W Song, Hee S Bae, Hyun M Doo, Ho S Yu, Kyu J Lee, Hee K Kim, Hyun Hwang, Geon Kwak, Daesik Kim, Seokjoong Kim, Young B Hong, Jung M Lee, Byung-Ok Choi

**Affiliations:** 1 Department of Health Sciences and Technology, SAIHST, Sungkyunkwan University, Seoul, 06351, Korea; 2 ToolGen, Inc., Seoul, 08501, Korea; 3 Department of Neurology, Samsung Medical Center, Sungkyunkwan University School of Medicine, Seoul, 06351, Korea; 4 Center for Genome Engineering, Institute for Basic Science (IBS), Seoul, 08826, Korea; 5 Department of Chemistry, Seoul National University, Seoul, 08826, Korea; 6 Department of Biochemistry, College of Medicine, Dong-A University, Busan 49201, Korea; 7 School of Life Science, Handong Global University, Pohang 37554, Korea

## Abstract

Charcot-Marie-Tooth 1A (CMT1A) is the most common inherited neuropathy without a known therapy, which is caused by a 1.4 Mb duplication on human chromosome 17, which includes the gene encoding the peripheral myelin protein of 22 kDa (*PMP22*). Overexpressed PMP22 protein from its gene duplication is thought to cause demyelination and subsequently axonal degeneration in the peripheral nervous system (PNS). Here, we targeted TATA-box of human *PMP22* promoter to normalize overexpressed *PMP22* level in C22 mice, a mouse model of CMT1A harboring multiple copies of human *PMP22*. Direct local intraneural delivery of CRISPR/Cas9 designed to target TATA-box of *PMP22* before the onset of disease, downregulates gene expression of *PMP22* and preserves both myelin and axons. Notably, the same approach was effective in partial rescue of demyelination even after the onset of disease. Collectively, our data present a proof-of-concept that CRISPR/Cas9-mediated targeting of TATA-box can be utilized to treat CMT1A.

## INTRODUCTION

Charcot-Marie-Tooth disease (CMT), which affects 1 in 2500 people, is the most common hereditary peripheral neuropathy (1). The predominant subtype, CMT type 1A (CMT1A), accounts for an estimated 50% of CMT cases, and involves prominent demyelination of the peripheral nerves (2). CMT1A is associated with a duplication of the gene encoding the peripheral myelin protein of 22 kDa (*PMP22*) (3,4). PMP22 is an intrinsic membrane protein of myelin that is developmentally induced in Schwann cells when initiating myelination in the peripheral nerve, and PMP22 overexpression causes a dedifferentiated phenotype in Schwann cells. On the other hand, deletion of the same 1.4 Mb region is also associated with other neuropathy, hereditary neuropathy with liability to pressure palsy (HNPP) (5). Transgenic rodents harboring multiple copies of *Pmp22* revealed that aberrant *Pmp22* expression is linked with CMT1A-associated peripheral neuropathy (6–9) whereas heterozygous knockout of *Pmp22* resembled some aspects of HNPP (10). These results indicate that proper regulation of *PMP22* is required to maintain PNS myelin. Therefore, a rational therapeutic approach for CMT1A is to downregulate *PMP22* expression in Schwann cells.

There have been attempts to reduce *PMP22* expression for CMT1A in indirect manners. For example, effective downregulation of *PMP22* by high-dose vitamin C in C22 mice led to clinical trials of vitamin C (11), which was ineffective in reducing *PMP22* gene expression level in skin biopsies from treated patients (12–14). Furthermore, although, progesterone antagonist was suggested as a potential therapy as it downregulated *Pmp22* gene in rodent models of *Pmp22* overexpression (15,16). However, as these approaches can influence in other cellular function, directly targeting *PMP22* itself would be more specific therapeutic approach. For direct targeting of PMP22, Zhao *et al.*, recently showed that direct downregulation of PMP22 via antisense oligonucleotide (ASO) can ameliorate the clinical symptoms associated with peripheral neuropathy in animal models of CMT1A. CRISPR-based gene editing also has great potential for treating genetic diseases including CMT1A by directly targeting disease causing genes. Additionally, CRISPR-based gene editing has potential to be a single dose therapy.

There are two different promoters driving expression of PMP22, P1 and P2. Transcribed mRNAs from these promoters only differ in their 5′ non-coding region (17,18). Although, *Pmp22* expression from P1 and P2 promoters are upregulated during myelination, *Pmp22* expression from P1 promoter is Schwann cell specific as transcripts from P2 promoter is expressed in other non-PNS tissues. For this reason, to downregulate *PMP22 in* Schwann Cell, we targeted TATA-box of *PMP22* P1 promoter as CRISPR-mediated TATA element disruption was shown to regulate mRNA expression (19).

Here, we tested the therapeutic efficacy of CRISPR/Cas9-mediated downregulation of *PMP22* via targeting TATA-box of *PMP22* P1 promoter region in the C22 mouse, a model of CMT1A by overexpressing human *PMP22* gene (20), allowing for direct translation of the target sequence to the clinic.

## MATERIALS AND METHODS

### Preparation of sgRNA

sgRNAs were generated by *in vitro* transcription using T7 polymerase (New England BioLabs) according to the manufacturer's protocol.

### Cell culture

A human Schwann-like cell line (ATCC crl-2885) and human Schwann cells (ScienCell) were maintained as described in the distributor's manual. Human Schwann-like cells were cultured in Dulbecco's Modified Eagle Medium (DMEM) with high glucose (WelGene) supplemented with 10% fetal calf serum (WelGene) and 1× penicillin/streptomycin (WelGene). Human Schwann cells were maintained in the provided Schwann Cell medium (ScienCell) and cultured on 1× poly-_D_-Lysine coated culture dishes. For differentiation, cells were cultured with DMEM with low glucose (WelGene) supplemented with 1% fetal calf serum (WelGene) and myelination signals, consisting of 100 ng/ml Nrg1 (Peprotech) and 100 μM dbcAMP (Sigma-Aldrich), for 7 days. For transfection of CRISPR/Cas9 components, ribonucleoprotein (RNP) complexes were formed by incubating 4 μg of Cas9-HA protein (ToolGen) with 1 μg of sgRNA at room temperature for 15 min. Then, 2 × 10^5^ cells were electroporated with the RNP complexes using a Neon electroporator (ThermoFisher) with 10 uL electroporation tips. For targeted deep sequencing, genomic DNA (gDNA) was collected from cells 72 h after transfection.

### *In vitro* primary Schwann cell culture, CRISPR/Cas9 transfection and DNA/RNA analysis

About 3–4 weeks old C22 mice (6–10 mice/preparation) were sacrificed by CO_2_ gas chamber. The accompanying procedure requires a sterile environment, equipment and cell culture tools. Both sciatic nerves were exposed, dissected out. Then, the surrounding membranes and muscular tissue of isolated nerves were carefully removed under a stereomicroscope. The epineurium was stripped off with fine forceps. The remaining nerves were then transferred to tube containing ice-cold phosphate-buffered saline (PBS) and centrifuged at 1500 rpm for 10 min. For single cell dissociation, enzymatic digestion was performed with 0.05% collagenase-A solution (Sigma) for 30 min at 37°C. Enzymatic activity was stopped by fetal bovine serum (Welgene) and centrifuged for 5 min at 1500 rpm. Dissociated cells were then seeded onto poly-L-ornithine- (Sigma) and laminin (ThermoFisher) coated dishes and allowed to adhere overnight. To eliminate contaminating fibroblasts, 10 μM AraC (Sigma) was added to the medium. After 48 h, the medium was replaced by DMEM (Welgene) containing 3% FBS with 3 μM forskolin (Sigma) and 20 ng/ml neuregulin (R&D systems) to expand the cells. For transfection of CRISPR/Cas9 components, 2 × 10^5^ cells were electroporated with the RNP complexes as described above using a Neon electroporator (ThermoFisher).

### Real time PCR (qRT-PCR)

#### Human Schwann cells in vitro

mRNA was extracted from primary human Schwann cells using a RNeasy mini kit (Qiagen) according to the manufacturer's protocol. A total of 100 ng of mRNA was then reverse transcribed using a High-capacity cDNA reverse transcription kit (ThermoFisher). Real-time quantitative reverse transcription polymerase chain reaction (qRT-PCR) was performed with 100 ng of cDNA using Taqman Gene expression master mix according to the manufacturer's protocol (Thermo Fisher) on QuantStudio 3. The level of *PMP22* expression was calculated using the C_T_ value and *GAPDH* was utilized as an endogenous control. Taqman probes (Thermo Fisher) utilized in this study are listed in Supplementary Table S7.

#### Primary Schwann cells from C22 mice

For gene expression analysis, total RNA were extracted using RNeasy Mini Kit (QIAGEN) according to manufacturer's protocol, 5 days post transfection. cDNA was obtained using SuperScript II according to the manufacturer's protocol (Thermo Fisher) as total mRNA extracted in the manner mentioned above. qRT-PCRwas performed using Power SYBR^®^ Green Master Mix (Thermo Fisher) protocol with the following primers:

Human P1-PMP22-F, 5′-CTTAGTCTGTCGGCTGCGGG-3′; Human P1-PMP22-R: 5′-GGCCAAACAGCGTAACCCCT-3′; Human P2-PMP22-F: 5′-CGTTAAAGGGGAACGCCAGGA-3′; Human P2-PMP22-R: 5′-CAGGGTGGCCTCAAACACAA-3′; Mouse Mpz-F: 5′-CGGACAGGGAAATCTATGGTGC-3′; Mouse Mpz-R: 5′-GCGCCAGGTAAAAGAGATGTCA-3′; Mouse P1-Pmp22-F: 5′-AGCTCCACCAGAGAACCTCTCA-3′; Mouse P1-Pmp22-R: 5′-TGAGGAGTAGCAGTGTTGGACGG-3′; Mouse P2-Pmp22-F: 5′- TGACCCGCAGCACAGCTGTCTTTG -3′; Mouse P2-Pmp22-R: 5′- TGAGGAGTAGCAGTGTTGGACGG 3′

#### Sciatic nerves in vivo

Total mRNA was extracted from sciatic nerves from WT and C22 mice 15 weeks post CRISPR/Cas9 administration, which had been treated with either *PMP22*-TATA or *mRosa26* RNPs, using an RNeasy Mini Kit (Qiagen). A total of 200 ng of total mRNA was then reverse transcribed using SuperScript™ II reverse transcriptase (Thermo Fisher). The resulting cDNA (3 μl) then served as the template for PCR amplification using the following primers: human PMP22-F, 5′-CCTCAGGAAATGTCCACCAC-3′; human PMP22-R, 5′-AGCACTCATCACGCACAGAC-3′; mouse actin-F, 5′-GTGACGTTGACATCCGTAAAGA-3′; and mouse actin-R, 5′-GCCGGACTCATCGTACTCTCC-3′. Real-time PCR reactions were performed in a total volume of 10 μl that consisted of 3 μl cDNA as template, 5 μl of SYBR Green PCR master mix (Applied Biosystems) and 2.5 pmol of each primer listed above. PCR amplifications (50 cycles at 95°C for 30 s and 60°C for 60 s) were performed using an ABI QuantStudio 6 Flex Real-Time PCR System (Applied Biosystems). To determine *PMP22* expression, the average Ct values calculated from quadruplicate PCR reactions were normalized to the average Ct values for actin. These normalized values were then used to calculate *PMP22* expression relative to the control according to formula 2^–(meanΔΔCt)^.

### Targeted deep sequencing

The on-target region was PCR amplified from gDNA extracted from transfected cells using Phusion polymerase (New England BioLabs). The resulting PCR amplicons were then subjected to paired-end deep sequencing using Mi-Seq (Illumina). Data from deep sequencing were analysed using the online Cas-Analyzer tool (www.rgenome.net) (21). Indels in the region 3 bp upstream from the protospacer-adjacent motif (PAM) sequence were considered to be mutations resulting from Cas9. Primer lists utilized in this study are listed in Supplementary Table S8.

### *In silico* identification of off-target sites

Potential off-target sites were identified *in silico* using an on-line tool (www.rgenome.net) (21). Sequences containing up to 3 bp mismatches were considered to be off-target sites.

### Digenome-seq

gDNA was purified from HeLa cells with a DNeasy Blood & Tissue Kit (Qiagen) according to the manufacturer's instructions. gDNA (10 μg) was mixed with the pre-incubated Cas9 protein (100 nM) and sgRNA (300 nM) RNP in a reaction volume of 1 ml (100 mM NaCl, 50 mM Tris–HCl, 10 mM MgCl2, 100 μg/ml bovine serum albumin, pH 7.9) and incubated at 37°C for 8 h. Digested gDNA was treated with RNase A (50 μg/ml) and purified again with a DNeasy Tissue Kit (Qiagen). A total of 1 μg of digested gDNA was fragmented using the Covaris system; the fragmented DNA was then ligated with adapters to produce libraries. These libraries were then subjected to whole genome sequencing using a HiSeq X Ten Sequencer (Illumina), at a sequencing depth of 30–40 × (Macrogen). An *in vitro* DNA cleavage score was calculated at each nucleotide position across the genome with a DNA cleavage scoring system described previously (22).

### Mice and intraneural injection

All animals were tested in a blinded manner. After administration, all genotyping, NCS data acquisition, and histological analyses were performed separately by different researchers without information regarding the treatments. All animal studies were carried out following the guidelines set by the Samsung Animal Care and Use Committee in accordance with the Association for Assessment and Accreditation of Laboratory Animal Care International regulations (SMC-20170206001).

The C22 mice [B6;CBACa-Tg(PMP22)C22Clh/H] were purchased from MRC Harwell (Oxfordshire, UK). Sciatic nerves of C22 and its wild-type littermate (WT) mice were treated with *mRosa26* or *PMP22*-TATA targeting RNPs at postnatal day 6 (p6) and 21 (p21). Intraneural injection was performed by a single injection distal to the sciatic notch as previously described (23,24). A 10 μl Hamilton syringe connected to a 33-gauge needle was used for intraneural injection. RNP complexes containing 11 μg of Cas9-HA protein (ToolGen) and 2.75 μg of sgRNA per animal with Lipofectamine 3000 (Thermo Fisher) were administered. To analyze the efficiency of CRISPR/Cas9-mediated genome editing efficiency and off-target analysis *in vivo*, gDNAs from sciatic nerves of treated animals 11 weeks post CRISPR/Cas9 administration were collected and subjected for targeted deep-sequencing.

### Immunohistochemistry and RNP distribution analysis *in vivo*

For immunostaining, mice were sacrificed 24 h post RNP administration. Sciatic nerves were isolated and fixed in 4% paraformaldehyde. These were then cryoprotected in 30% sucrose and processed for cryosection. Longitudinal sections were permeabilized with cold methanol and washed with PBS. Sections were then blocked and permeabilized with 10% normal goat serum and 0.3% Triton X-100 in PBS for 1 h at room temperature. Rabbit Anti-HA tag (Cell signaling Technology C29F4) were applied on sections for 24 h at 4°C followed by anti-rabbit Alexa-Fluor 488 (ThermoFisher Scientific) for 1 h at room termperatures. Sections were then mounted with ProLong Gold Antifade Mountant with DAPI (ThermoFisher Scientific). Images were taken under a Axio Scan.Z1 fluorescence microscope with Zen software (Carl Zeiss).

### Electrophysiological study

To assess electrophysiological status, a nerve conduction study (NCS) was performed for WT and C22 mice 11 weeks post CRISPR/Cas9 administration as previously described (25). Briefly, mice were anesthetized with 1.5% isoflurane supplied using a nose cone for the duration of the procedure. The fur from the distal back to the hind limbs was completely removed. The NCS was performed using a Nicolet VikingQuest device (Natus Medical). For measurement of motor nerve conduction velocity, stimulating cathodes were placed at sciatic notch and 6 mm distal to the sciatic notch, while recording electrodes were placed on the muscle belly of the tibialis anterior muscle. And a ground electrode was placed at the animal's back, near the midline. Motor nerve conduction velocity (MNCV) and compound muscle action potential (CMAP) amplitudes were determined by an independent examiner who was blinded for the genotype and treatment groups. We measured CMAPs at supramaximal stimulation.

### Nerve histology and imaging

The sciatic nerves were biopsied from both WT and C22 mice 11 weeks post CRISPR/Cas9 administration and pathological examinations of affected specimens were performed with microscopic analyses. Specimens were fixed overnight with 2.5% glutaraldehyde in 4% paraformaldehyde solution at 4 C. After incubation for 1 h in 1% OsO_4_, the specimens were dehydrated in an ethanol series, passed through propylene oxide, and embedded in epoxy resin (Epok 812, 02–1001, Oken, Japan). The tissues were cut into semi-thin (1 μm) sections (2 mm piece cut from sciatic notch where intraneural injection was performed) and stained with toluidine blue for 5 to 10 s. Semi-thin sections were imaged using Bx51 upright microscope (Olympus) and analyzed using Cell sense (Olympus). Ultrathin sections (65 nm) were collected on 200 mesh nickel grids and stained for 15 min in 2% uranyl acetate and 5 min lead citrate. The specimens were observed with a Hitachi HT7700 electron microscope at 80 kV. Electron microscope analysis was performed by the electron microscope facility of the Samsung Biomedical Research Institute. About 500 axons were counted per animal. The numbers of myelinated axons and unmyelinated axons (axons greater than 1 μm in diameter without a myelin sheath) were expressed as percentage of total counted axons. And, onion bulbs were also expressed as percentage of total counted axons. Axons with a diameter larger than 1 μm were counted from randomly selected 20–25 images from each animal (50 × 40 μm, *n* = 5). Determination of the *g*-ratio (axon diameter divided by fiber diameter) was performed by measuring the outer and inner diameter of the myelin sheath using the Zeiss Zen2 program (Carl Zeiss).

### Gastrocnemius muscle examination and measurement

Biopsies were taken from the gastrocnemius muscles of WT or C22 mice 15 weeks post CRISPR/Cas9 treatments. The animals were sacrificed by CO_2_ inhalation. The gastrocnemius muscle was detached and weighed to compare the gastrocnemius muscle weight (mg) with the total body weight (g).

### Statistical analysis

**Figure 1.**
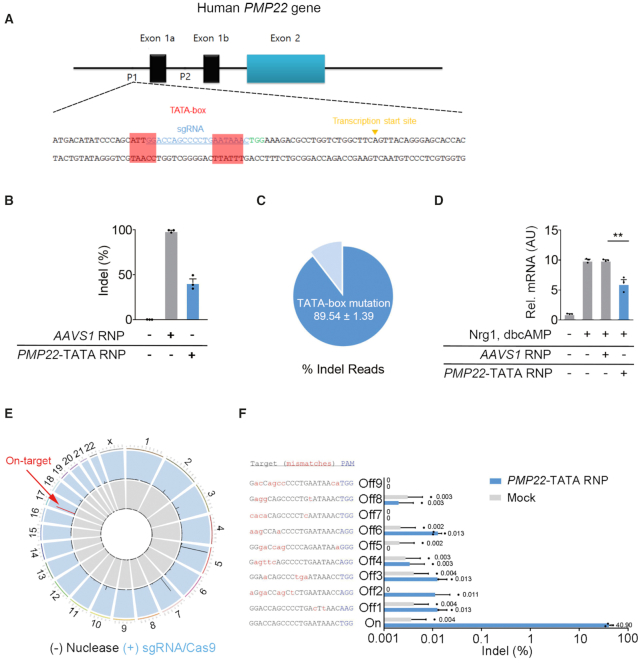
Efficient and specific downregulation of *PMP22* via CRISPR/Cas9 targeting the TATA-box region of the human *PMP22* gene *in vitro*. (**A**) The target sequence in the promoter region of the human *PMP22* locus. TATA-box sequences are shown in red boxes and the PAM and sgRNA target sequences are shown in green and blue, respectively. (**B**) Indel frequencies measured by targeted deep sequencing in primary human Schawann cells. (**C**) Frequency of sequences with TATA-box mutations relative to the total number of sequences with indels (of (B)) as determined by targeted deep sequencing (*n* = 3). (**D**) Relative *PMP22* mRNA levels in primary human Schwann cells with or without myelination signals (Nrg1, dbcAMP) and RNP complexes, measured by quantitative real-time PCR (qRT-PCR) (*n* = 3, ** *P* < 0.01). (**E**) Genome-wide Circos plot showing *in vitro* cleavage sites. Human genomic DNA is shown in gray and *PMP22-*TATA RNP digested genomic DNA is shown in blue. (**F**) Off-target sites validated in human Schwann cells by targeted deep sequencing. The mismatched nucleotides are shown in red and PAM sequences are shown in blue. Error bars indicate SEM.

All values are expressed as mean ± SEM. The statistical significance of the data presented in Figure 1D was evaluated by one-way ANOVA with post-hoc Tukey's multiple comparison. The statistical significance of data presented in elsewhere were evaluated by student's t-test. Graphs and data generated in this study were analyzed using GraphPad Prism. The level of significance was set to 0.05.

## RESULTS

### CRISPR/Cas9-mediated genome editing of TATA-box of *PMP22*

To target TATA-box region of human *PMP22*, we searched for PAM sequences (NGG for *Streptococcus pyogenes* Cas9) near the TATA-box region of human *PMP22* and designed eight different single guide RNAs (sgRNAs) to target this region (Supplementary Table S1). The activities of these sgRNAs were screened after transfection of sgRNA-Cas9 RNP complexes (26) into a human cell line derived from metastatic site of neurofibromatosis type 1 displaying Schwann cell-like characteristics (human Schwann-like cell line) (Supplementary Figure S1). Cas9-mediated insertion or deletion (indel) mutations at the desired sites were evaluated using targeted deep sequencing. We found that sgRNA1, gRNA4 and sgRNA7 showed high indel efficiency. From these sgRNAs we selected sgRNA1 as lead sgRNA as it showed greatest efficiency to disrupt the TATA-box from targeted deep sequencing (Data not shown), we selected this sgRNA (*PMP22*-TATA sgRNA hereafter) as the lead sgRNA. Bioinformatics analysis revealed that the selected PMP22-TATA sgRNA sequence is conserved in both human and mouse. The high efficiency of *PMP22*-TATA sgRNA in indel mutation formation was replicated in primary human Schwann cells (Figure 1A and B). Targeted deep sequencing analysis revealed that 89.54 ± 1.39% of the total indel reads corresponded to the intended TATA-box region of human *PMP22* (Figure 1C and Supplementary Table S2).

### Targeted *in vitro* deletion of TATA-box of *PMP22* downregulates *PMP22* expression level

To determine whether mutations in the *PMP22* TATA-box lead to a reduction in human *PMP22* gene expression, we performed qRT-PCR analysis. Because *PMP22* is transcribed during the late stage of Schwann cell differentiation, we treated primary human Schwann cells with a known potent pro-myelination signal, consisting of Neuregulin-1 (Nrg1) and dibutryryl cyclic AMP (dbcAMP) (27), for 7 days, which resulted in an ∼9 fold induction of human *PMP22* expression compared to cells without Nrg1 and dbcAMP treatment (Figure 1D). In contrast, the *PMP22*-TATA RNP treated culture showed an ∼6 fold induction of human *PMP22* expression (Figure 1D). Schwann cells treated with a control *AAVS1*-targeting RNP before exposure to the differentiation signal showed no difference in human *PMP22* gene expression when compared to a culture treated with the differentiation signal alone (Figure 1D).

As *PMP22*-TATA RNP targets *PMP22* P1 transcript, we evaluated its targeting specificity. For this, we treated *PMP22*-TATA or *mRosa26* targeting RNP in primary Schwann cells isolated from C22 mice. Successful targeting of TATA-box of P1 promoter of human and mouse *PMP22* locus was confirmed by targeted deep sequencing (Supplementary Figure S2a). To determine specific targeting of P1 transcript of *PMP22* we performed qRT-PCR using primers specific for human P1-*PMP22* (P1-*hPMP22*), P2-*PMP22* (P2-*hPMP22*), mouse P1-*Pmp22* (P1-*mPmp22*) and mouse P2-*Pmp22* (P2-*mPmp22*). In *PMP22*-TATA RNP treated culture, significant knockdown of P1-*hPMP22* transcript and although not statistically significant, knockdown trend of P1-*mPmp22* transcript were found whereas no changes in neither P2-*hPMP22* nor P2-*mPmp22* expression level when compared to *mRosa26* RNP treated culture (Supplementary Figure S2b–e). These results indicate that PMP22-TATA RNP is highly specific to P1 transcript of *PMP22*.

### No detectable off-target effects in genome edited cells

To analyze the specificity of the *PMP22*-TATA RNP, we performed *in silico*-based off-target analysis, which showed potential off-target sites containing up to 3 bp mismatches in the human genome. Targeted deep sequencing revealed no indel mutations above sequencing error rates (0.1%, on average) at the potential off-target sites that were found by *in silico* analysis (Supplementary Figure S3 and Table S3). Because *in silico*-based off-target analysis can be biased (28), we performed Digenome-seq (22), an unbiased whole genome sequencing-based off-target analysis. This approach revealed nine potential off-target sites cleaved by the *PMP22*-TATA RNP *in vitro*. However, re-analysis by targeted deep sequencing detected no aberrant indel mutations in these potential off-target sites *in vivo* (Figure 1E and F; Supplementary Table S4). Together, these results indicate that efficient and specific disruption of the *PMP22* TATA-box by the *PMP22*-TATA RNP can modulate the level of *PMP22* transcription in primary Schwann cells.

### Establishment of intraneural CRISPR-RNP delivery to sciatic nerve and evaluation of its distribution

**Figure 2.**
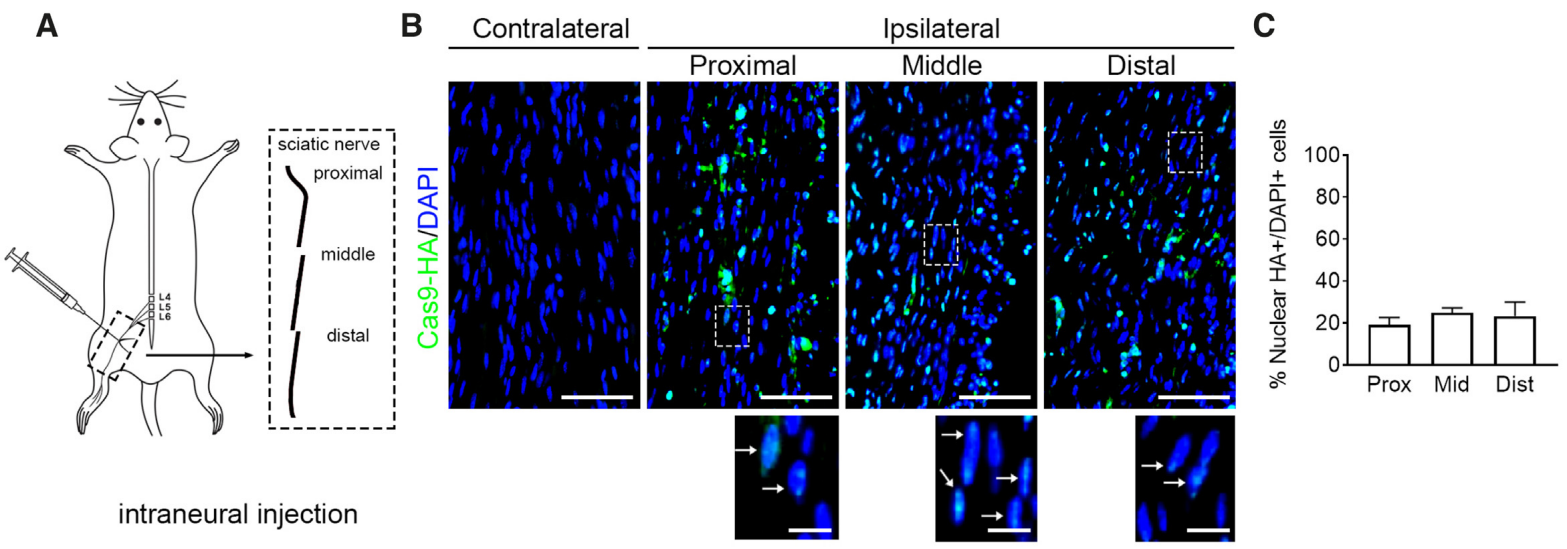
Intraneural CRISPR-RNP delivery results in efficient biodistribution of RNP *in vivo*. (**A**) Schematic diagram showing the intraneural injection into the sciatic nerve immediately distal to the sciatic notch along with the three segments (proximal, middle and distal) of the sciatic nerves utilized for analysis. (**B**) Representative immunostained images from contralateral (uninjected) and proximal, middle and distal segments of ipsilateral sciatic nerves (Scale bar = 50 μm). Higher magnification images of ipsilateral nerves showing nuclear localization of Cas9-HA were shown below (Scale bar = 10 μm). (**C**) Percentage of DAPI-positive nuclei containing HA-positive Cas9 (*n* = 3 nerves and mice).

For PNS genome editing *in vivo*, we delivered CRISPR-RNP comprising sgRNA targeting mouse Rosa26 (*mRosa26*) and Cas9 with HA tag (Cas9-HA) encapsulated with liposome to left sciatic nerves (ipsilateral) of WT C57Bl/6 mice at postnatal day 6 (p6). The other uninjected sciatic nerves (contralateral) were utilized as control. Specifically, we injected RNP immediately distal to the sciatic notch as described before (Figure 2A) (23,24). As preassembled CRISPR-RNP was shown to degrade within 3 days *in vivo* (29), we sacrificed animals 24 h post-injection and prepared cryosections of injected sciatic nerves for RNP distribution. For this, we immunostained sections for HA and counterstained with DAPI to visualize nuclei. To determine RNP spread along the whole sciatic nerve, we divided the nerve into three segments (proximal, middle and distal) and evaluated HA-positive RNP containing DAPI+ nuclei (Figure 2B). We found that the percentages of HA-positive nuclei were ∼20% in the proximal, middle and distal segments (Figure 2C), indicating injected RNP was distributed evenly throughout the nerve and localized into the nucleus for gene editing.

### Specific *in vivo* genome editing of TATA-box of *PMP22*

**Figure 3.**
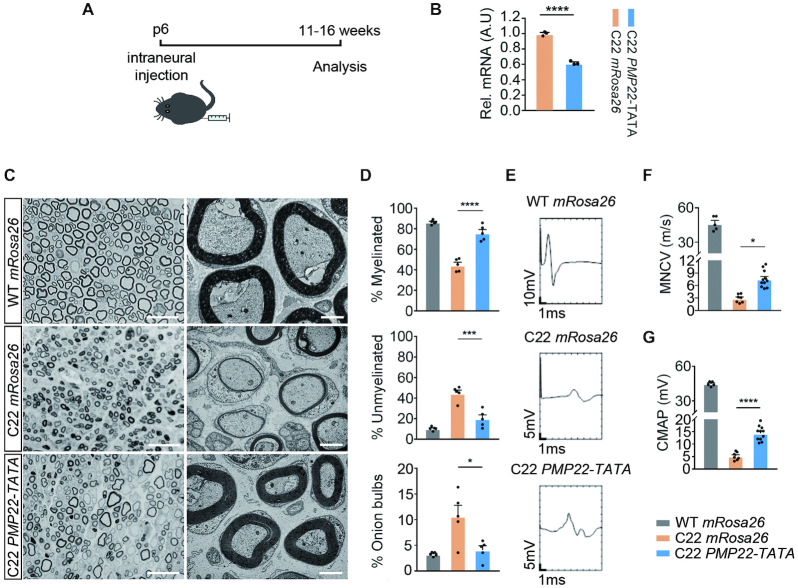
CRISPR/Cas9-mediated *PMP22* downregulation prevented disease phenotypes in CMT1A mice. (**A**) Six-day-old C22 pups were injected with mRosa26 RNP or PMP22-TATA RNP intraneurally, and then culled at 11–16 weeks of age for analysis. (**B**) Relative *PMP22* mRNA levels from *mRosa26* or *PMP22*-TATA RNP treated sciatic nerves from C22 mice, measured by qRT-PCR (*n* = 3 for both treatments). (**C**) Representative semi-thin cross-section images stained with toluidine blue (left) and ultra-thin electron micrographs from same sections (right) from WT mice injected with *mRosa26* RNP and C22 mice injected with *mRosa26* or *PMP22*-TATA RNP. Scale bar = 20μm for semi-thin and for 2 μm ultra-thin sections. (**D**) Quantification of percentage of myelinated, unmyelinated and onion bulb axons (*n* = 5). (**E**) Representative electrophysiological trace, (**F**) measurement of motor nerve conduction velocity (MNCV) and (**G**) compound muscle action potentials (CMAP) from WT mice injected with *mRosa26* RNP and C22 mice injected with *mRosa26* or *PMP22*-TATA RNP. *n* = 7 for *mRosa26* RNP; *n* = 11 *PMP22*-TATA RNP. *n* = 7 for *mRosa26* RNP, *n* = 10 for *PMP22*-TATA RNP. *, *P* < 0.05, **, *P* < 0.01. ***, *P* < 0.005, **** *P* < 0.0001.

Having achieved efficient intraneural delivery of CRISPR-RNP, we then tested whether the *PMP22*-TATA RNP can regulate *PMP22* transcription *in vivo*. We delivered RNP complexes encapsulated with liposome carrying sgRNAs targeting either *mRosa26* as a control or *PMP22*-TATA into the left sciatic nerve (ipsilateral) of C22 mice or their wild-type littermates (WT) by intraneural injection at p6 (Figure 3A). The right sciatic nerve was used as an internal control (contralateral). We examined the gene editing efficiency of RNP complexes after intraneural delivery via targeted deep sequencing of genomic DNA collected from whole sciatic nerves 11 weeks post injection, which showed ∼11% indel formation at the appropriate locus in both *mRosa26* and *PMP22*-TATA RNP treated sciatic nerves (Supplementary Figure S4a and Table S5). To ascertain whether the TATA-box mutations downregulated human *PMP22* expression *in vivo*, we performed qRT-PCR analysis of mRNA extracted from whole *PMP22*-TATA RNP treated sciatic nerves 16 weeks post injection. In agreement with the *in vitro* analysis, we found that human *PMP22* gene expression was significantly reduced (44.64% ± 11.41 knockdown) when compared to controls (Figure 3B).

We then investigated whether the *PMP22*-TATA RNP caused any off-target mutations *in vivo*. To address this question, we performed *in silico*-based off-target analysis and identified eight potential off-target sites containing up to 3 bp mismatches in the mouse genome (Supplementary Table S6). Targeted deep sequencing revealed no comparable indel mutations at these sites in *PMP22*-TATA RNP treated ipsilateral nerves when compared to contralateral nerves (Supplementary Figure S5).

### Prevention of neuropathological and electrophysiological deficits in C22 mice following targeted genome editing of TATA-box of *PMP22* at p6

To investigate whether *PMP22*-TATA RNP-mediated reduction of *PMP22* transcription prevents demyelination in C22 mice, we performed histological analysis of the semi-thin cross sections of the sciatic nerves treated with either *PMP22*-TATA or *mRosa26* RNPs 11 weeks post injection and revealed significant reduction of unmyelinated axons and ‘onion bulbs’ (Supplementary Figure S6), a classic hallmark of repetitive segmental demyelination and remyelination of peripheral nerve (Figure 3C and D). We also measured the axon diameter and fiber diameter (axon with myelin) to determine the *g*-ratio, and found that it was reduced in *PMP22*-TATA compared to *mRosa26* RNP treated nerve, which suggests that a thicker myelin sheath formed in *PMP22*-TATA compared to *mRosa26* RNP treated nerve (Supplementary Figure S7). Furthermore, we also observed more large diameter axons in *PMP22*-TATA compared to *mRosa26* RNP treated nerve (Figure 3C and Supplementary Figure S7).

Given the substantial improvements seen in histological analysis of myelination, we interrogated the electrophysiological profiles of both groups. We found a significantly increased motor nerve conduction velocity (MNCV; Figure 3E and F) in *PMP22*-TATA compared to *mRosa26* treated sciatic nerves of C22 mice 10 weeks post injection, which correlates with the increment in myelin thickness and axon diameter in *PMP22*-TATA RNP nerves. Moreover, *PMP22*-TATA RNP treated nerve showed a significant increase in the amplitude of compound muscle action potential (CMAP; Figure 3E and G). Furthermore, along with histological and electrophysiological improvements, we found a significant muscle gain in *PMP22*-TATA compared to *mRosa26* RNP treated C22 mice at 15 weeks post administration of RNPs (Supplementary Figure S8).

### Neuropathological and electrophysiological rescue of C22 mice following targeted genome editing of TATA-box of *PMP22* at p21

**Figure 4.**
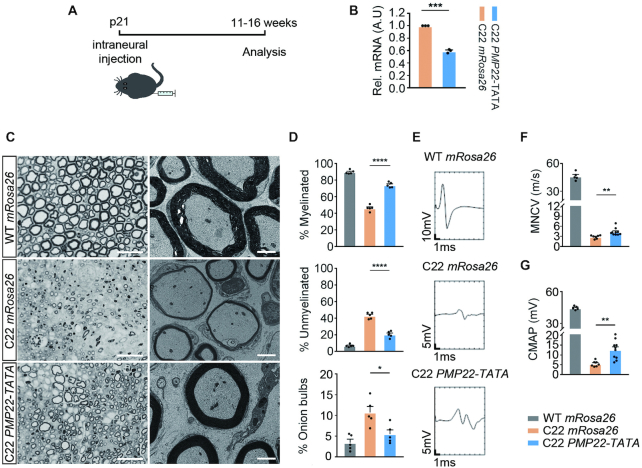
CRISPR/Cas9-mediated *PMP22* downregulation ameliorated disease phenotypes in CMT1A mice. (**A**) Twenty-one-day-old C22 mice were injected with mRosa26 RNP or PMP22-TATA RNP intraneurally, and then culled 11–16 weeks of age for analysis. (**B**) Relative *PMP22* mRNA levels from *mRosa26* or *PMP22*-TATA RNP-treated sciatic nerves from C22 mice, measured by qRT-PCR (*n* = 3 for both treatments). (**C**) Representative semi-thin cross-section images stained with toluidine blue (left) and ultra-thin electron micrographs from same sections (right) from WT mice injected with *mRosa26* RNP and C22 mice injected with *mRosa26* or *PMP22*-TATA RNP. Scale bar = 20 μm for semi-thin and for 2 μm ultra-thin sections. (**D**) Quantification of percentage of myelinated, unmyelinated and onion bulb axons (*n* = 5). (**E**) Representative electrophysiological trace, (**F**) measurement of motor nerve conduction velocity (NCV) and (**G**) compound muscle action potentials (CMAP) from WT mice injected with *mRosa26* RNP and C22 mice injected with *mRosa26* or *PMP22*-TATA RNP. *n* = 7 for *mRosa26* RNP; *n* = 10 *PMP22*-TATA RNP. *n* = 7 for *mRosa26* RNP, *n* = 10 for *PMP22*-TATA RNP. *, *P* < 0.05, **, *P* < 0.01. ***, *P* < 0.005, **** *P* < 0.0001

Although we have found a significant amelioration of demyelination-associated neuropathy in C22 mice by targeted mutagenesis of TATA-box of *PMP22*, administration of therapeutic CRISPR at p6 could be a prevention rather than treatment. To see whether our approach can modulate phenotypes after the onset of disease in C22 mice, we administered PMP22-TATA RNP at p21 intraneurally (Figure 4A) at which C22 mice exhibit neuropathological symptoms including histopathology and electrophysiology (30). In line with the prevention study (administration at p6), we achieved similar downregulation of human *PMP22* measured by qRT-PCR analysis when compared to *mRosa26* treated controls (40.67% ± 1.74 knockdown; Figure 4B). Downregulation of *PMP22* by PMP22-TATA RNP partially rescued histopathological phenotypes in C22 mice where less number of unmyelinated axons and ‘onion bulbs’ whereas increased number of myelinated axons were found in sciatic nerve sections of PMP22-TATA RNP treated C22 mice when compared to mRosa26 treated C22 mice control (Figure 4C and D). Furthermore, improved histopathological features correlated with partial rescue of electrophysiological properties where increased MNCV and CMAP were found in sciatic nerves of PMP22-TATA RNP treated C22 mice when compared to mRosa26 treated C22 mice control (Figure 4E–G).

## DISCUSSION

Overexpressed *PMP22* in Schwann cells due to its duplication in CMT1A patients has been generally thought to be the cause of peripheral neuropathy including demyelination in affected patients. As heterozygous deletion of *PMP22* (leads to downregulation of *PMP22)* cause HNPP (31), a rationale therapeutic approach for CMT1A would be normalizing *PMP22* gene expression to physiological level.

Transcription of *PMP22* is regulated by multiple elements such as EGR2 and SOX10 transcription factor responsive intronic enhancer region (32), the late myelination Schwann cell-specific element (LMSE) (33) and super-enhancer region located far distal from the *PMP22* promoter (34,35). These multiple regulatory components of *PMP22* gene transcription further support the importance of tight regulation of its expression.

It was shown that targeted TATA-box editing of *c-myc* gene by CRISPR/Cas9 reduced its mRNA and subsequently protein expression (19). We utilized similar approach and for specific downregulation of *PMP22* in Schwann cells, TATA-box of *PMP22* P1 promoter was targeted by CRISPR/Cas9. In contrast to targeting protein coding sequence of PMP22, TATA-box editing may prevent unwanted effects from knocking down PMP22 expression too low which may cause HNPP. *PMP22*-TATA targeting CRISPR/Cas9 treatment resulted in specific editing of TATA-box of *PMP22* P1 promoter as validated by qRT-PCR-based gene expression analysis using specific primers for P1 and P2 transcripts of *PMP22*. Schwann cells were shown to express *PMP22* driven by either P1 or P2 promoter whereas non-neural cells express *PMP22* driven by P2 promoter specifically (18). Thereby, PMP22-TATA sgRNA would have reduced potential off-target in non-Schwann cells. Furthermore, unbiased whole genome sequencing followed by targeted deep sequencing revealed no genome-wide off-target cleavage, suggesting that selected lead sgRNA targeting TATA-box of *PMP22* in this study is potentially translatable.

There are some intriguing results that need to be discussed. We observed ∼40% reduction in *PMP22* transcripts in sciatic nerves but detected indels in only ∼10% of sequenced *PMP22* transgenes. It is difficult to speculate the reason behind this without further study; however, CRISPR/Cas9-mediated *in vivo* gene editing studies also reported phenotypic changes that exceeds on-target indel frequency (36,37). In addition, it was suprising that the *PMP22* downregulation effects appear to be quite consistent throughout the *in vivo* experiments. Although we achieved quite consistent indel efficiency *in vivo*, each CRISPR delivery thought to produce different indel formation in each cells. To explain the consistent downregulation of PMP22 *in vivo*, a clonal analysis with different indels of the TATA-box can be performed in the future to delineate quantitative downregulation of PMP22 upon different indels of the TATA-box. Furthermore, although *PMP22*-TATA RNP treatment showed phenotypic correction when compared to *mRosa26* targeting RNP, this never reached wild-type level. To further enhance therapeutic efficacy, more efficient delivery vehicles are required for the PNS and intrathecal administration of certain viruses may pave the way for this (38,39).

Furthermore, although it varies, Schwann cells from CMT1A patients exhibit ∼1.7-fold PMP22 overexpression. Whereas, C22 mice harbors seven–eight copies of human *PMP22*, which display an ∼2.7-fold *PMP22* mRNA overexpression when compared to endogenous mouse *Pmp22* (8,11), which indicates that C22 mouse model is not a perfect model for CMT1A as it harbors high copy number of PMP22 and the expression level from transgene differs from CMT1A patient scenario. Therefore, transgenic animal models with low copy number of *PMP22* such as C3 mice or *Pmp22* transgenic rats which harbors four copies of human *PMP22* or three copies of mouse *Pmp22*, respectively could be utilized to determine therapeutic efficacy of our strategy in more CMT1A-like *in vivo* scenario.

Notably, we observed therapeutic efficacy of TATA-box of *PMP22* P1 promoter targeted CRISPR/Cas9 in both p6 and p21 timepoints. As previous studies observed a prominent neurological phenotype as early as p21 in c22 mice (30), our study demonstrates that targeting TATA-box of PMP22 P1 promoter by CRISPR/Cas9 not only applicable in preventative manner before demyelination (p6 timepoint), but also may applicable in treatment paradigm (p21 timepoint). Further investigations are required to test whether targeting TATA-box of *PMP22* by CRISPR/Cas9 also has therapeutic effect in later stage of development, which would help determining therapeutic time window for our approach.

To our surprise, single dose of liposomal *PMP22*-TATA RNP delivery resulted in the partial phenotypic rescue of C22 mice. Through our analysis, we found that delivered RNPs were evenly distributed along the sciatic nerves, however only ∼20% of cells contained RNPs within their nucleus which may suggest relatively low indel efficiency (∼11% at p6) and partial not full rescue of phenotypes of C22 mice *in vivo*.

Although RNP-based delivery of CRISPR/Cas9 showed efficient gene editing *in vivo*, one should consider about immunogenicity and stability of the Cas9 protein in the host. To overcome potential immunogenicity against Cas9 protein, proper encapsulation of RNP complexes using nanoparticles required. On the other hand, the rational Cas9 engineering approach to eliminate T-cell epitopes showed promise to evade immunogenicity against Cas9 protein (40). To increase the stability of the Cas9 protein in the host, other variants of Cas9 such as Cas9 derived from a thermophilic bacterium, Geobacillus stearothermophilus (GeoCas9) can be utilized (41).

Moreover, to further evaluate therapeutic efficacy of targeting TATA-box of *PMP22* promoter *in vivo*, systemic delivery approach to maximize PNS Schwann cell targeting efficiency such as intrathecal administration using viral vectors maybe required (42). Furthermore, this systemic delivery would allow us to investigate on behavioural improvement upon therapeutic vehicle delivery.

For successful translation of CRISPR/Cas9-based genome editing for therapy, potential unwanted off-target effects should be thoroughly considered. For this, we performed whole-genome sequencing based Digenome-seq to study off-target effects. However, this may not be enough to cover other off-target effects that are recently identified such as unintended large deletions and complex rearrangements (43). Although there are differences regarding developmental stage of target cells (Schwann cells versus mouse induced pluripotent stem cells) and modality of CRISPR/Cas9 tools (RNP versus piggybac) between current study and (43), meticulous analysis of off-target effects that could be caused by CRISPR/Cas9 should be evaluated.

While we were preparing this study, therapeutic approach involving ASO-based downregulation of *PMP22* was reported to be effective in rodent models of CMT1A when delivered in repeated dose (44). In consistent with ASO-based approach, our results indicate that lowering *PMP22* level has potential to be translated into the clinic. Moreover, as CRISPR/Cas9 can edit DNA, it has the advantage of potentially a single dose regimen.

In summary, our data demonstrate the proof-of-principle for *PMP22* downregulation via CRISPR/Cas9 targeting TATA-box of the *PMP22* promoter region. Non-viral delivery of *PMP22*-TATA RNP directly into the sciatic nerves of C22 mice ameliorates the neuropathological symptoms caused by *PMP22* over-expression. Therefore, CRISPR/Cas9-mediated modification of the TATA-box of *PMP22* promoter holds promise for the treatment of CMT1A. Furthermore, to our knowledge, apart from being the first study to demonstrate genome editing in the PNS, this is the first study of utilization of CRISPR/Cas9 to treat gene duplication mutation and further study warrants for the use of CRISPR/Cas9 for other gene duplication mutations.

## DATA AVAILABILITY

The datasets generated or analyzed during the current study are available from the corresponding author on reasonable request.

## Supplementary Material

gkz1070_Supplemental_File

## References

[1] Skre H. Genetic and clinical aspects of Charcot-Marie-Tooth's disease. Clin. Genet. 1974; 6:98–118. 4430158 10.1111/j.1399-0004.1974.tb00638.x

[2] Boerkoel C.F., Takashima H., Garcia C.A., Olney R.K., Johnson J., Berry K., Russo P., Kennedy S., Teebi A.S., Scavina M. et al. Charcot-Marie-Tooth disease and related neuropathies: mutation distribution and genotype-phenotype correlation. Ann. Neurol. 2002; 51:190–201. 11835375 10.1002/ana.10089

[3] Lupski J.R., de Oca-Luna R.M., Slaugenhaupt S., Pentao L., Guzzetta V., Trask B.J., Saucedo-Cardenas O., Barker D.F., Killian J.M., Garcia C.A. et al. DNA duplication associated with Charcot-Marie-Tooth disease type 1A. Cell. 1991; 66:219–232. 1677316 10.1016/0092-8674(91)90613-4

[4] Raeymaekers P., Timmerman V., Nelis E., De Jonghe P., Hoogendijk J.E., Baas F., Barker D.F., Martin J.J., De Visser M., Bolhuis P.A. et al. Duplication in chromosome 17p11.2 in Charcot-Marie-Tooth neuropathy type 1a (CMT 1a). The HMSN Collaborative Research Group. Neuromuscul. Disord. 1991; 1:93–97. 1822787 10.1016/0960-8966(91)90055-w

[5] Chance P.F., Alderson M.K., Leppig K.A., Lensch M.W., Matsunami N., Smith B., Swanson P.D., Odelberg S.J., Disteche C.M., Bird T.D. DNA deletion associated with hereditary neuropathy with liability to pressure palsies. Cell. 1993; 72:143–151. 8422677 10.1016/0092-8674(93)90058-x

[6] Sereda M., Griffiths I., Puhlhofer A., Stewart H., Rossner M.J., Zimmerman F., Magyar J.P., Schneider A., Hund E., Meinck H.M. et al. A transgenic rat model of Charcot-Marie-Tooth disease. Neuron. 1996; 16:1049–1060. 8630243 10.1016/s0896-6273(00)80128-2

[7] Magyar J.P., Martini R., Ruelicke T., Aguzzi A., Adlkofer K., Dembic Z., Zielasek J., Toyka K.V., Suter U. Impaired differentiation of Schwann cells in transgenic mice with increased PMP22 gene dosage. J. Neurosci. 1996; 16:5351–5360. 8757248 10.1523/JNEUROSCI.16-17-05351.1996 PMC6578876

[8] Huxley C., Passage E., Manson A., Putzu G., Figarella-Branger D., Pellissier J.F., Fontes M. Construction of a mouse model of Charcot-Marie-Tooth disease type 1A by pronuclear injection of human YAC DNA. Hum. Mol. Genet. 1996; 5:563–569. 8733121 10.1093/hmg/5.5.563

[9] Perea J., Robertson A., Tolmachova T., Muddle J., King R.H., Ponsford S., Thomas P.K., Huxley C. Induced myelination and demyelination in a conditional mouse model of Charcot-Marie-Tooth disease type 1A. Hum. Mol. Genet. 2001; 10:1007–1018. 11331611 10.1093/hmg/10.10.1007

[10] Adlkofer K., Frei R., Neuberg D.H., Zielasek J., Toyka K.V., Suter U. Heterozygous peripheral myelin protein 22-deficient mice are affected by a progressive demyelinating tomaculous neuropathy. J. Neurosci. 1997; 17:4662–4671. 9169527 10.1523/JNEUROSCI.17-12-04662.1997 PMC6573352

[11] Passage E., Norreel J.C., Noack-Fraissignes P., Sanguedolce V., Pizant J., Thirion X., Robaglia-Schlupp A., Pellissier J.F., Fontes M. Ascorbic acid treatment corrects the phenotype of a mouse model of Charcot-Marie-Tooth disease. Nat. Med. 2004; 10:396–401. 15034573 10.1038/nm1023

[12] Lewis R.A., McDermott M.P., Herrmann D.N., Hoke A., Clawson L.L., Siskind C., Feely S.M., Miller L.J., Barohn R.J., Smith P. et al. High-dosage ascorbic acid treatment in Charcot-Marie-Tooth disease type 1A: results of a randomized, double-masked, controlled trial. JAMA Neurol. 2013; 70:981–987. 23797954 10.1001/jamaneurol.2013.3178 PMC3752369

[13] Nobbio L., Visigalli D., Radice D., Fiorina E., Solari A., Lauria G., Reilly M.M., Santoro L., Schenone A., Pareyson D. et al. PMP22 messenger RNA levels in skin biopsies: testing the effectiveness of a Charcot-Marie-Tooth 1A biomarker. Brain. 2014; 137:1614–1620. 24812204 10.1093/brain/awu071

[14] Pareyson D., Reilly M.M., Schenone A., Fabrizi G.M., Cavallaro T., Santoro L., Vita G., Quattrone A., Padua L., Gemignani F. et al. Ascorbic acid in Charcot-Marie-Tooth disease type 1A (CMT-TRIAAL and CMT-TRAUK): a double-blind randomised trial. Lancet. Neurol. 2011; 10:320–328. 21393063 10.1016/S1474-4422(11)70025-4 PMC3154498

[15] Sereda M.W., Meyer zu Horste G., Suter U., Uzma N., Nave K.A. Therapeutic administration of progesterone antagonist in a model of Charcot-Marie-Tooth disease (CMT-1A). Nat. Med. 2003; 9:1533–1537. 14608378 10.1038/nm957

[16] Meyer zu Horste G., Prukop T., Liebetanz D., Mobius W., Nave K.A., Sereda M.W. Antiprogesterone therapy uncouples axonal loss from demyelination in a transgenic rat model of CMT1A neuropathy. Ann. Neurol. 2007; 61:61–72. 17262851 10.1002/ana.21026

[17] Bosse F., Zoidl G., Wilms S., Gillen C.P., Kuhn H.G., Muller H.W. Differential expression of two mRNA species indicates a dual function of peripheral myelin protein PMP22 in cell growth and myelination. J. Neurosci. Res. 1994; 37:529–537. 8021974 10.1002/jnr.490370412

[18] Suter U., Snipes G.J., Schoener-Scott R., Welcher A.A., Pareek S., Lupski J.R., Murphy R.A., Shooter E.M., Patel P.I. Regulation of tissue-specific expression of alternative peripheral myelin protein-22 (PMP22) gene transcripts by two promoters. J. Biol. Chem. 1994; 269:25795–25808. 7929285

[19] Slobodin B., Han R., Calderone V., Vrielink J., Loayza-Puch F., Elkon R., Agami R. Transcription impacts the efficiency of mRNA translation via Co-transcriptional N6-adenosine methylation. Cell. 2017; 169:326–337. 28388414 10.1016/j.cell.2017.03.031 PMC5388891

[20] Huxley C., Passage E., Robertson A.M., Youl B., Huston S., Manson A., Saberan-Djoniedi D., Figarella-Branger D., Pellissier J.F., Thomas P.K. et al. Correlation between varying levels of PMP22 expression and the degree of demyelination and reduction in nerve conduction velocity in transgenic mice. Hum. Mol. Genet. 1998; 7:449–458. 9467003 10.1093/hmg/7.3.449

[21] Park J., Lim K., Kim J.S., Bae S. Cas-analyzer: an online tool for assessing genome editing results using NGS data. Bioinformatics. 2017; 33:286–288. 27559154 10.1093/bioinformatics/btw561 PMC5254075

[22] Kim D., Bae S., Park J., Kim E., Kim S., Yu H.R., Hwang J., Kim J.I., Kim J.S. Digenome-seq: genome-wide profiling of CRISPR-Cas9 off-target effects in human cells. Nat. Methods. 2015; 12:237–243. 25664545 10.1038/nmeth.3284

[23] Gonzalez S., Fernando R.N., Perrin-Tricaud C., Tricaud N. In vivo introduction of transgenes into mouse sciatic nerve cells in situ using viral vectors. Nat. Protoc. 2014; 9:1160–1169. 24762783 10.1038/nprot.2014.073

[24] Sargiannidou I., Kagiava A., Bashiardes S., Richter J., Christodoulou C., Scherer S.S., Kleopa K.A. Intraneural GJB1 gene delivery improves nerve pathology in a model of X-linked Charcot-Marie-Tooth disease. Ann. Neurol. 2015; 78:303–316. 26010264 10.1002/ana.24441

[25] Lee J., Jung S.C., Joo J., Choi Y.R., Moon H.W., Kwak G., Yeo H.K., Lee J.S., Ahn H.J., Jung N. et al. Overexpression of mutant HSP27 causes axonal neuropathy in mice. J. Biomed. Sci. 2015; 22:43. 26141737 10.1186/s12929-015-0154-y PMC4490621

[26] Kim S., Kim D., Cho S.W., Kim J., Kim J.S. Highly efficient RNA-guided genome editing in human cells via delivery of purified Cas9 ribonucleoproteins. Genome Res. 2014; 24:1012–1019. 24696461 10.1101/gr.171322.113 PMC4032847

[27] Arthur-Farraj P., Wanek K., Hantke J., Davis C.M., Jayakar A., Parkinson D.B., Mirsky R., Jessen K.R. Mouse schwann cells need both NRG1 and cyclic AMP to myelinate. Glia. 2011; 59:720–733. 21322058 10.1002/glia.21144 PMC5722196

[28] Chuai G.H., Wang Q.L., Liu Q. In silico meets in vivo: towards computational CRISPR-Based sgRNA Design. Trends Biotechnol. 2017; 35:12–21. 27418421 10.1016/j.tibtech.2016.06.008

[29] Kim K., Park S.W., Kim J.H., Lee S.H., Kim D., Koo T., Kim K.E., Kim J.H., Kim J.S. Genome surgery using Cas9 ribonucleoproteins for the treatment of age-related macular degeneration. Genome Res. 2017; 27:419–426. 28209587 10.1101/gr.219089.116 PMC5340969

[30] Verhamme C., King R.H., ten Asbroek A.L., Muddle J.R., Nourallah M., Wolterman R., Baas F., van Schaik I.N. Myelin and axon pathology in a long-term study of PMP22-overexpressing mice. J. Neuropathol. Exp. Neurol. 2011; 70:386–398. 21487305 10.1097/NEN.0b013e318217eba0

[31] van Paassen B.W., van der Kooi A.J., van Spaendonck-Zwarts K.Y., Verhamme C., Baas F., de Visser M. PMP22 related neuropathies: Charcot-Marie-Tooth disease type 1A and hereditary neuropathy with liability to pressure palsies. Orphanet. J. Rare Dis. 2014; 9:38. 24646194 10.1186/1750-1172-9-38 PMC3994927

[32] Jones E.A., Lopez-Anido C., Srinivasan R., Krueger C., Chang L.W., Nagarajan R., Svaren J. Regulation of the PMP22 gene through an intronic enhancer. J. Neurosci. 2011; 31:4242–4250. 21411665 10.1523/JNEUROSCI.5893-10.2011 PMC3100536

[33] Maier M., Castagner F., Berger P., Suter U. Distinct elements of the peripheral myelin protein 22 (PMP22) promoter regulate expression in Schwann cells and sensory neurons. Mol. Cell Neurosci. 2003; 24:803–817. 14664827 10.1016/s1044-7431(03)00246-x

[34] Lopez-Anido C., Poitelon Y., Gopinath C., Moran J.J., Ma K.H., Law W.D., Antonellis A., Feltri M.L., Svaren J. Tead1 regulates the expression of Peripheral Myelin Protein 22 during Schwann cell development. Hum. Mol. Genet. 2016; 25:3055–3069. 27288457 10.1093/hmg/ddw158 PMC5181599

[35] Pantera H., Moran J.J., Hung H.A., Pak E., Dutra A., Svaren J. Regulation of the neuropathy-associated Pmp22 gene by a distal super-enhancer. Hum. Mol. Genet. 2018; 27:2830–2839. 29771329 10.1093/hmg/ddy191 PMC6077802

[36] Long C., McAnally J.R., Shelton J.M., Mireault A.A., Bassel-Duby R., Olson E.N. Prevention of muscular dystrophy in mice by CRISPR/Cas9-mediated editing of germline DNA. Science. 2014; 345:1184–1188. 25123483 10.1126/science.1254445 PMC4398027

[37] Ran F.A., Cong L., Yan W.X., Scott D.A., Gootenberg J.S., Kriz A.J., Zetsche B., Shalem O., Wu X., Makarova K.S. et al. In vivo genome editing using Staphylococcus aureus Cas9. Nature. 2015; 520:186–191. 25830891 10.1038/nature14299 PMC4393360

[38] Kagiava A., Sargiannidou I., Theophilidis G., Karaiskos C., Richter J., Bashiardes S., Schiza N., Nearchou M., Christodoulou C., Scherer S.S. et al. Intrathecal gene therapy rescues a model of demyelinating peripheral neuropathy. Proc. Natl. Acad. Sci. U.S.A. 2016; 113:E2421–E2429. 27035961 10.1073/pnas.1522202113 PMC4855595

[39] Kagiava A., Karaiskos C., Richter J., Tryfonos C., Lapathitis G., Sargiannidou I., Christodoulou C., Kleopa K.A. Intrathecal gene therapy in mouse models expressing CMT1X mutations. Hum. Mol. Genet. 2018; 27:1460–1473. 29462293 10.1093/hmg/ddy056

[40] Ferdosi S.R., Ewaisha R., Moghadam F., Krishna S., Park J.G., Ebrahimkhani M.R., Kiani S., Anderson K.S. Multifunctional CRISPR-Cas9 with engineered immunosilenced human T cell epitopes. Nat. Commun. 2019; 10:1842. 31015529 10.1038/s41467-019-09693-x PMC6478683

[41] Harrington L.B., Paez-Espino D., Staahl B.T., Chen J.S., Ma E., Kyrpides N.C., Doudna J.A. A thermostable Cas9 with increased lifetime in human plasma. Nat. Commun. 2017; 8:1424. 29127284 10.1038/s41467-017-01408-4 PMC5681539

[42] Kagiava A., Kleopa K.A. Intrathecal delivery of viral vectors for gene therapy. Methods Mol. Biol. 2018; 1791:277–285. 30006718 10.1007/978-1-4939-7862-5_22

[43] Kosicki M., Tomberg K., Bradley A. Repair of double-strand breaks induced by CRISPR-Cas9 leads to large deletions and complex rearrangements. Nat. Biotechnol. 2018; 36:765–771. 30010673 10.1038/nbt.4192 PMC6390938

[44] Zhao H.T., Damle S., Ikeda-Lee K., Kuntz S., Li J., Mohan A., Kim A., Hung G., Scheideler M.A., Scherer S.S. et al. PMP22 antisense oligonucleotides reverse Charcot-Marie-Tooth disease type 1A features in rodent models. J. Clin. Invest. 2018; 128:359–368. 29202483 10.1172/JCI96499 PMC5749515

