# Supplementary material for: Targeted PMP22 TATA-box editing by CRISPR/Cas9 reduces demyelinating neuropathy of Charcot-Marie-Tooth disease type 1A in mice
Source: Nucleic Acids Res. 2019 Nov 12;48(1):130–40. doi: 10.1093/nar/gkz1070 (PMC7145652; doi:10.1093/nar/gkz1070)
Supplement: gkz1070_Supplemental_File [file gkz1070_supplemental_file.docx]

**Supplementary Table 1. sgRNAs used in this study.**

| **Target site** | **sgRNA #** | **Target sequence - PAM (5' to 3')** |
| --- | --- | --- |
| Human *PMP22-*TATA | sgRNA 1 | GGACCAGCCCCTGAATAAAC-TGG |
|  | sgRNA 2 | GGCGTCTTTCCAGTTTATTC-AGG |
|  | sgRNA 3 | GCGTCTTTCCAGTTTATTCA-GGG |
|  | sgRNA 4 | CGTCTTTCCAGTTTATTCAG-GGG |
|  | sgRNA 5 | TTCAGGGGCTGGTCCAATGC-TGG |
|  | sgRNA 6 | TCAGGGGCTGGTCCAATGCT-GGG |
|  | sgRNA 7 | ACCATGACATATCCCAGCAT-TGG |
|  | sgRNA 8 | TTTCCAGTTTATTCAGGGGC-TGG |
| Mouse *Rosa26* | sgRNA 1 | GGCGGTCCTCAGAAGCCAGG-AGG |
| Human *AAVS1* | sgRNA 1 | ATGGAGCCAGAGAGGATCCT-GGG |

**Supplementary Table 2. Representative targeted deep-sequencing reads (5 most frequent indel patterns) from *PMP22*-TATA RNP treated human primary Schwann cell culture. The TATA-box is shown in red, the PAM sequence in green, and the sgRNA sequence is underlined.**

| **Indel** | **Local Sequence** | **Frequency (%)** |
| --- | --- | --- |
| WT | ACTGAAGCCAGACCAGGCGTCTTTCCAG**TTTATT**CAGGGGCTGGTCCAATGCTGGGATATGTCATGGTGGCCTGAGAGGTTCTCAGCCTC |  |
| -1 | ACTGAAGCCAGACCAGGCGTCTTTCCAG-TTATTCAGGGGCTGGTCCAATGCTGGGATATGTCATGGCGGCCTGAGAGGTTCTCAGCCTC | 14.00 |
| -2 | ACTGAAGCCAGACCAGGCGTCTTTCCAGTT--TTCAGGGGCTGGTCCAATGCTGGGATATGTCATGGTGGCCTGAGAGGTTCTCAGCCTC | 8.86 |
| +1 | ACTGAAGCCAGACCAGGCGTCTTTCCAGTTTTATTCAGGGGCTGGTCCAATGCTGGGATATGTCATGGCGGCCTGAGAGGTTCTCAGCCTC | 3.84 |
| -3 | ACTGAAGCCAGACCAGGCGTCTTTCCAGT---TTCAGGGGCTGGTCCAATGCTGGGATATGTCATGGCGGCCTGAGAGGTTCTCAGCCTC | 3.53 |
| -4 | ACTGAAGCCAGACCAGGCGTCTTTCCAG----TTCAGGGGCTGGTCCAATGCTGGGATATGTCATGGCGGCCTGAGAGGTTCTCAGCCTC | 2.81 |

**Supplementary Table 3. Potential off-target sites of the *PMP22*-TATA targeting sgRNA1 in the human genome (GRCh38.hg38) revealed by *in silico* analysis.**

|  | **Location** | **Position** | **Target sequence (5' to 3')** | **Gene** |
| --- | --- | --- | --- | --- |
| On-target | chr17 | 15265347 | GGACCAGCCCCTGAATAAACTGG | *hPMP22*-TATA |
| Off1 | chr5 | 135488419 | GGACCAGCCaCaGAATAAACAAG | Intergenic |
| Off2 | chr8 | 140735957 | tGACCAGtCCaTGAATAAACCAG | PTK2 (Intron) |
| Off3 | chr12 | 14124312 | GGACCAGaCaCTGAATAtACCAG | Intergenic |
| Off4 | chr4 | 97775621 | GGACCAGCCaCaGAATAAAtTGG | STPG2 (Intron) |
| Off5 | chr5 | 26531145 | GGAtCAGCCCCaGAATAAAtTAG | Intergenic |
| Off6 | chr1 | 41780482 | GGAgCAtCCCCaGAATAAACCAG | HIVEP3 (Intron) |
| Off7 | chr1 | 157564675 | GGAtCAGCgtCTGAATAAACAAG | Intergenic |
| Off8 | chr13 | 20254256 | aGACCAGCCCCaGAAcAAACAAG | Intergenic |
| Off9 | chr15 | 100401183 | GtACgAGCCCCTGAATAAAtAGG | CERS3 (Exon) |
| Off10 | chr6 | 26006396 | GGACCAaaCaCTGAATAAACCAG | Intergenic |
| Off11 | chr20 | 10136908 | GcACCAGCCaCTGAATtAACAAG | SNAP25 (Intron) |
| Off12 | chrX | 7525146 | GtACCAGCCaCTGAAaAAACAGG | Intergenic |
| Off13 | chr18 | 1972251 | GaACCAGCCCCTGAtTAgACCAG | Intergenic |
| Off14 | chr18 | 77536261 | GtACCAGCCaCTGAAaAAACAGG | Intergenic |
| Off15 | chr11 | 30065750 | GtACCAGCCCCTGcAaAAACAGG | Intergenic |
| Off16 | chr11 | 30579429 | GcACCAGgCCtTGAATAAACAAG | MPP2D2 (Intron) |
| Off17 | chr11 | 35726323 | GGcCCAGCCaCTGAgTAAACTAG | TRIM44 (Intron) |
| Off18 | chr11 | 112468286 | GGAattGCCCCTGAATAAACAAG | RP11-65M17.3 (Intron) |

**Supplementary Table 4. Potential off-target sites of the *PMP22*-TATA targeting sgRNA1 in the human genome (GRCh38.hg38) revealed by Digenome-seq. Mismatched nucleotides are shown in red and PAM sequences in blue.**

|  | **Location** | **Position** | **Target sequence (5' to 3')** | **Gene** |
| --- | --- | --- | --- | --- |
| On-target | chr17 | 15265347 | GGACCAGCCCCTGAATAAACTGG | *hPMP22*-TATA |
| Off1 | chr5 | 38420810 | GGgaacagCCCTGAATAAACCTG | *EGFLAM* (Intron) |
| Off2 | chr7 | 28618099 | aGgaCCagCtCTGAATAACCAGG | *CREB5* (Intron) |
| Off3 | chr5 | 38420811 | GGAaCAGCCCtgaATAAACCTGG | *EGFLAM* (Intron) |
| Off4 | chr10 | 93462291 | GagttcAGCCCCTGAATAACAGG | Intergenic |
| Off5 | chr3 | 78627344 | GGgaCcagCCCCAGAATAAaGGG | Intergenic |
| Off6 | chr2 | 131586033 | aagCCAaCCCCTGAATAAACAGG | Intergenic |
| Off7 | chr18 | 56254369 | cacaCAGCCCCTcAATAAACTGG | *ALPK2* (Intron) |
| Off8 | chr22 | 27477459 | GaggCAGCCCCTGtATAAACTGG | Intergenic |
| Off9 | chr6 | 91586787 | GacCagccCCCTGAATAAcaTGG | Intergenic |

**Supplementary Table 5. Representative targeted deep-sequencing reads (5 most frequent indel patterns) from sciatic nerves from C22 mice after administration of the *PMP22*-TATA RNP. The TATA-box is shown in red, the PAM sequence in green, and the sgRNA sequence is underlined.**

| **Indel** | **Local Sequence** | **Frequency (%)** |
| --- | --- | --- |
| WT | ACTGAAGCCAGACCAGGCGTCTTTCCAG**TTTATT**CAGGGGCTGGTCCAATGCTGGGATATGTCATGGTGGCCTGAGAGGTTCTCAGCCTC |  |
| -1 | ACTGAAGCCAGACCAGGCGTCTTTCCAG-TTATTCAGGGGCTGGTCCAATGCTGGGATATGTCATGGTGGCCTGAGAGGTTCTCAGCCTC | 1.56 |
| -9 | ACTGAAGCCAGACCAGGCGTCTTTC---------CAGGGGCTGGTCCAATGCTGGGATATGTCATGGTGGCCTGAGAGGTTCTCAGCCTC | 1.53 |
| +1 | ACTGAAGCCAGACCAGGCGTCTTTCCAGTTTTATTCAGGGGCTGGTCCAATGCTGGGATATGTCATGGTGGCCTGAGAGGTTCTCAGCCTC | 1 |
| -4 | ACTGAAGCCAGACCAGGCGTCTTTCCAG----TTCAGGGGCTGGTCCAATGCTGGGATATGTCATGGTGGCCTGAGAGGTTCTCAGCCTC | 0.8 |
| -10 | ACTGAAGCCAGACCAGGCGTCT----------TTCAGGGGCTGGTCCAATGCTGGGATATGTCATGGTGGCCTGAGAGGTTCTCAGCCTC | 0.4 |

**Supplementary Table 6. Potential off-target sites of the *PMP22*-TATA targeting sgRNA1 in the mouse genome (GRCm38.mm10) revealed by *in silico* analysis.**

|  | **Location** | **Position** | **Target sequence (5' to 3')** | **Gene** |
| --- | --- | --- | --- | --- |
| On-target | chr17 | 15265347 | GGACCAGCCCCTGAATAAACTGG | hPMP22-TATA |
| Off1 | chr12 | 118558427 | GtACCAGCCCCTGAcaAAACAGG | Intergenic |
| Off2 | chr1 | 74579514 | GGAgCAGCCCCgGAATgAACAGG | Zfp142 (Exon) |
| Off3 | chr13 | 50187695 | GGACCAGCCCCTGtATAccCTGG | Intergenic |
| Off4 | chr13 | 50319559 | GGACCAGCCCCTGtATAccCTGG | Intergenic |
| Off5 | chr13 | 50623450 | GGACCAGCCCCTGtATAccCTGG | Intergenic |
| Off6 | chr2 | 29191358 | GGcCCtGCCCCTaAATAAACAGG | Intergenic |
| Off7 | chr9 | 102823783 | GGAtCAGCCCCaGAATAAcCTGG | Intergenic |
| Off8 | chrX | 101405421 | GGACtAGCCCCTGAgTAcACTGG | Zmym3 (Exon) |

**Supplementary Table 7. Accession number of taqman probes used for qRT-PCR**

| **Target Gene** | **Taqman Gene Experssion Assay** | **Accession number** |
| --- | --- | --- |
| *PMP22* | Hs00165556_m1 | NM_000304.3 |
| *GAPDH* | HS02786624_g1 | NM_001256799.2 |

**Supplementary Table 8. Primers used in this study.**

|  | **Target site** | **Primer-F (5' to 3')** | **Primer-R (5' to 3')** |
| --- | --- | --- | --- |
| On-Target | *hPMP22*-TATA | CACAGGGCAGTCAGAGACCC | GCAAACAAAGTTGGACACTG |
|  | *mRosa26* | AGACTCCCGCCCATCTTCTAGAAA | AAGTCGCTCTGAGTTGTTATCAGT |
|  | *AAVS1* | CAGTGAAACGCACCAGACG | AATCTGCCTAACAGGAGGTG |
| Off-target  (*In silico, in vitro*) | *hPMP22*-TATA Off1 | GAGGGAATGGGGACCAAAGGCATT | TCATGTGGGGTGATGTTCAGGAAG |
|  | *hPMP22*-TATA Off2 | AGAGCAGCTGACCTGAGGTCCAA | CCCAAGGGTAGAGTGCAAGTAAAC |
|  | *hPMP22*-TATA Off3 | GCATCCTAGCTCATTTGGTCTGCT | GAGAGGATTCCTCATGAATGGGAT |
|  | *hPMP22*-TATA Off4 | ACCAAACACTACACTTGGTTACTG | CTCCCACTAGCAATTTTAAAGTCT |
|  | *hPMP22*-TATA Off5 | GAATGTTCAGCACAGGTTTCCTTG | GGTCAAAAGGAGCTCCATATTTGA |
|  | *hPMP22*-TATA Off6 | CAGGACACCCATGGCCAAATCCAG | CAGAGCCTCCTGCAGGGATGTCAA |
|  | *hPMP22*-TATA Off7 | GCCTGCCAAGGTGACTCTCATCTA | TGCCCAGGCTGATCTTGAACTCCT |
|  | *hPMP22*-TATA Off8 | CCCAGAGTTAAGAGGTTCTTTCCT | GAAGCTACTCCAGTGCAACTAGCT |
|  | *hPMP22*-TATA Off9 | ACGCAGTCTGTTCTGTGCAGTGT | AGGCCTTCCCAAGGAAGACCCTGA |
|  | *hPMP22*-TATA Off10 | GCTGATCACTGGCCAAATCCAGCT | GGGAAACAATGGGATCAAGCTGCA |
|  | *hPMP22*-TATA Off11 | GCCCCTTTGTAAGTTGAGGAGCAT | CCCTCTACCTCTCTCAATGGGCTT |
|  | *hPMP22*-TATA Off12 | CAGACAAGCAAATGCTGAGAGATT | CCTGTCATTATGATGTTCGCTAGT |
|  | *hPMP22*-TATA Off13 | CCAGAGTTGGCCTCCTACAGAGAT | GTGGATGCCCCACTACTGTTCATT |
|  | *hPMP22*-TATA Off14 | TACCCAATTTGCCAGTCTGTGTCT | ACCACCAGGCCTGCCCTACAAGA |
|  | *hPMP22*-TATA Off15 | TGTGAATTTGATCCTGGCATTATG | TACAGACAAGCAGATGCTGAGAGA |
|  | *hPMP22*-TATA Off16 | CAGTCAACAGAGCTCTAACCTCCT | AGCACCTGGTTGCACATCAACTT |
|  | *hPMP22*-TATA Off17 | CATGTGGTCCCTGAACGTGAATGA | GTCTGTCGCTTGCCCTCTTCTCT |
|  | *hPMP22*-TATA Off18 | ATGCAGGGCCTCTAGACCATTTCA | CTCAGCCCTTTGTGCACTCACCT |
| Off-target (*Digenome-seq,*  *in vitro*) | *hPMP22*-TATA Off1 | TGCACATCGCAAACATTTCG | TGGGTATCGCACTGTGTCAG |
|  | *hPMP22*-TATA Off2 | AGGTTCACATGGCTTGTGGT | ATATCTGAAATGCCCGCAGG |
|  | *hPMP22*-TATA Off3 | TGCACATCGCAAACATTTCG | TGGGTATCGCACTGTGTCAG |
|  | *hPMP22*-TATA Off4 | TCTTTAAAGGCCTTATCTCC | TTCTGCTTGAGAATTCATCC |
|  | *hPMP22*-TATA Off5 | CTCCTAATCTTTCACTTAGG | CAAAGCCTGGTATAACATAG |
|  | *hPMP22*-TATA Off6 | TCACTTCGAGCATCTGTGG | CCAAATGACAGGCTGAGCT |
|  | *hPMP22*-TATA Off7 | AGCAGGAAGTGAAGGCTAAG | ATGTAACGTGGCAACTCTGG |
|  | *hPMP22*-TATA Off8 | GTGTTGCTCTCGTCAATTAG | AGGTGTTGTACATGGAGAAG |
|  | *hPMP22*-TATA Off9 | TGTGAGCCACCATACCCAGC | CCTGCAGTCCTTTGCGGATC |
| Off-target  (*In silico, In vivo*) | *hPMP22*-TATA Off1 | TCGCTGCCAGTATAACATGC | AACTCCAGTCTCTAGACTCG |
|  | *hPMP22*-TATA Off2 | AATAGTTTGACGTTGGAGCC | ACTCCCAACATGTTCTCCTG |
|  | *hPMP22*-TATA Off3 | ATCATCGCTCACAGAGTCC | ACGACTGCAGGATCTTAATG |
|  | *hPMP22*-TATA Off4 | TGGATGGAGGTTGGGAATCC | TTGAGGCAGCAGCACTCTCC |
|  | *hPMP22*-TATA Off5 | AGTCTATCCTAGCAGCTCC | ACTGAGACCAGATAATGCAG |
|  | *hPMP22*-TATA Off6 | AAGAGATGCGAGTTGTTCC | CCTCTTCTACTCTGAGTGG |
|  | *hPMP22*-TATA Off7 | ACCTGGTTTATCACAAGCTA | AACGTGAACAGAAGGATTTC |
|  | *hPMP22*-TATA Off8 | ATCACTCCATCAGAGTCAGG | TGGCTCCTTCTATTCTCTCC |


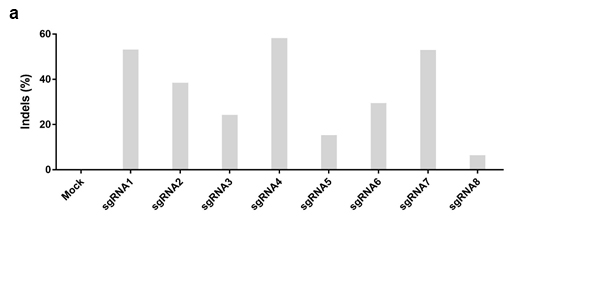


**Supplementary Fig. 1 Screening of sgRNA sequences for targeting the human *PMP22* TATA box**

(a) Indel frequencies associated with each sgRNA measured by targeted deep sequencing in a human Schwann-like cell line.


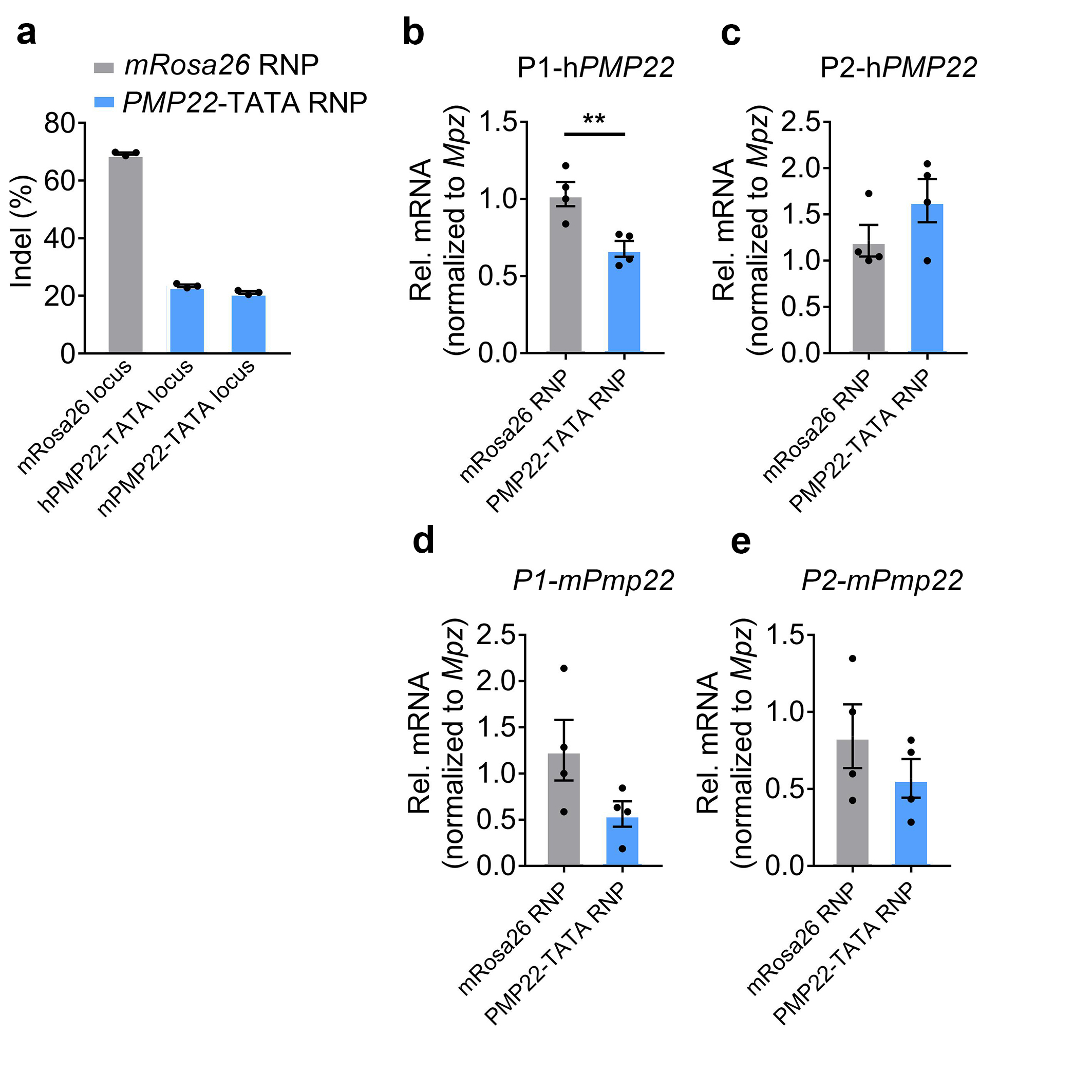


**Supplementary Fig. 2 Confirmation of P1-PMP22 targeting specificity of PMP22-TATA RNP**

Specific targeting of P1-*PMP22* by *PMP22*-TATA was evaluated in C22 primary Schwann cells. (a) Indel frequencies at either human or mouse PMP22 TATA-box locus measured by targeted deep sequencing in C22 primary Schwann cells treated with *mRosa26* or *PMP22*-TATA RNP complexes. qRT-PCR analyses of (b) human P1-*PMP22*, (c) human P2-*PMP22*, (d) mouse P1-*Pmp22* and (e) mouse P2-*Pmp22* (*n* = 3-4 for both treatments).

**
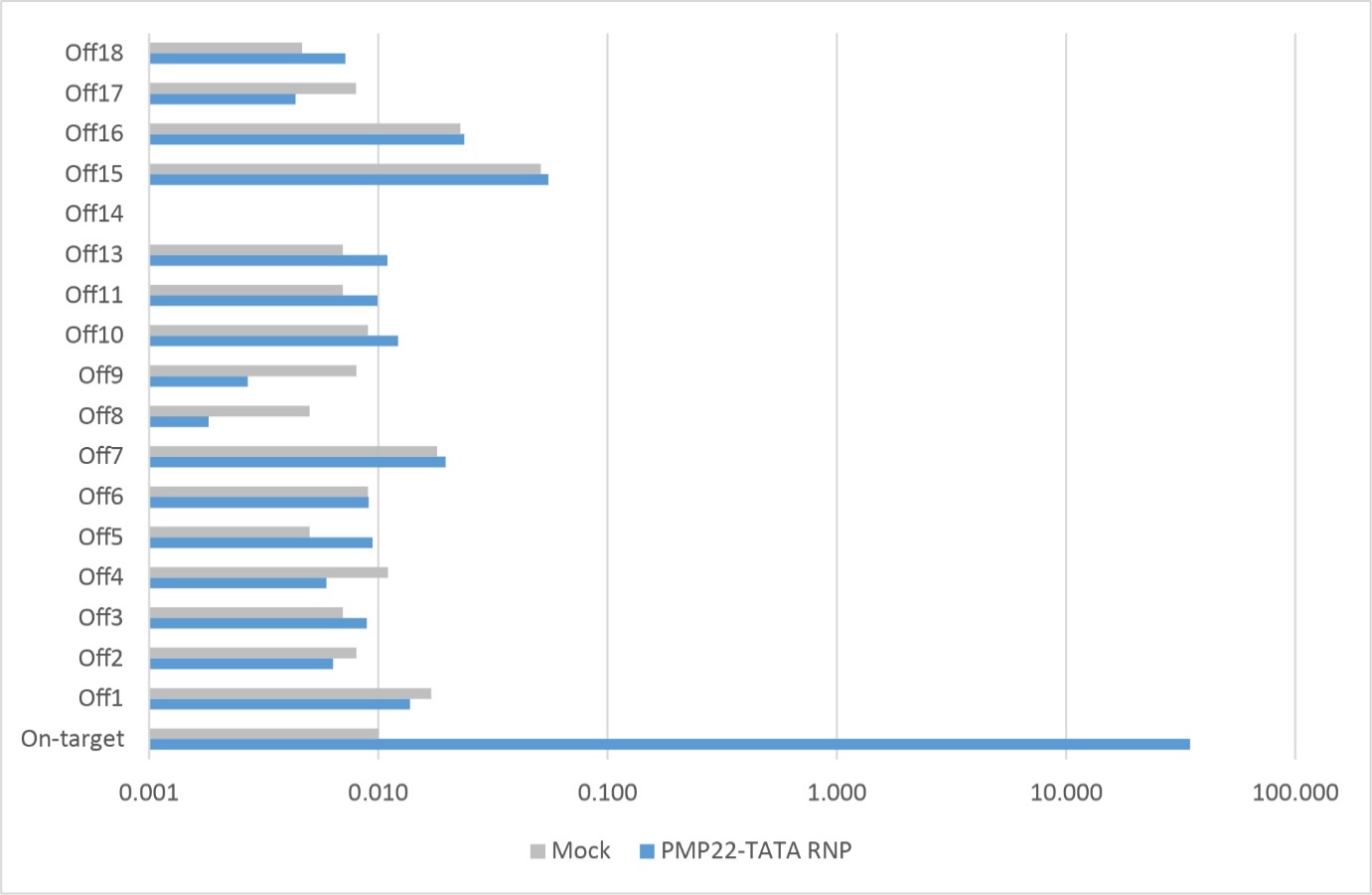
**

**Supplementary Fig. 3 *In silico* off-target analysis of PMP22-TATA RNP in primary human Schwann cells**

Indel frequencies in primary human Schwann cells, determined by targeted deep sequencing, at *PMP22*-TATA RNP on-target and off-target sites identified by *in silico* off-target analysis.


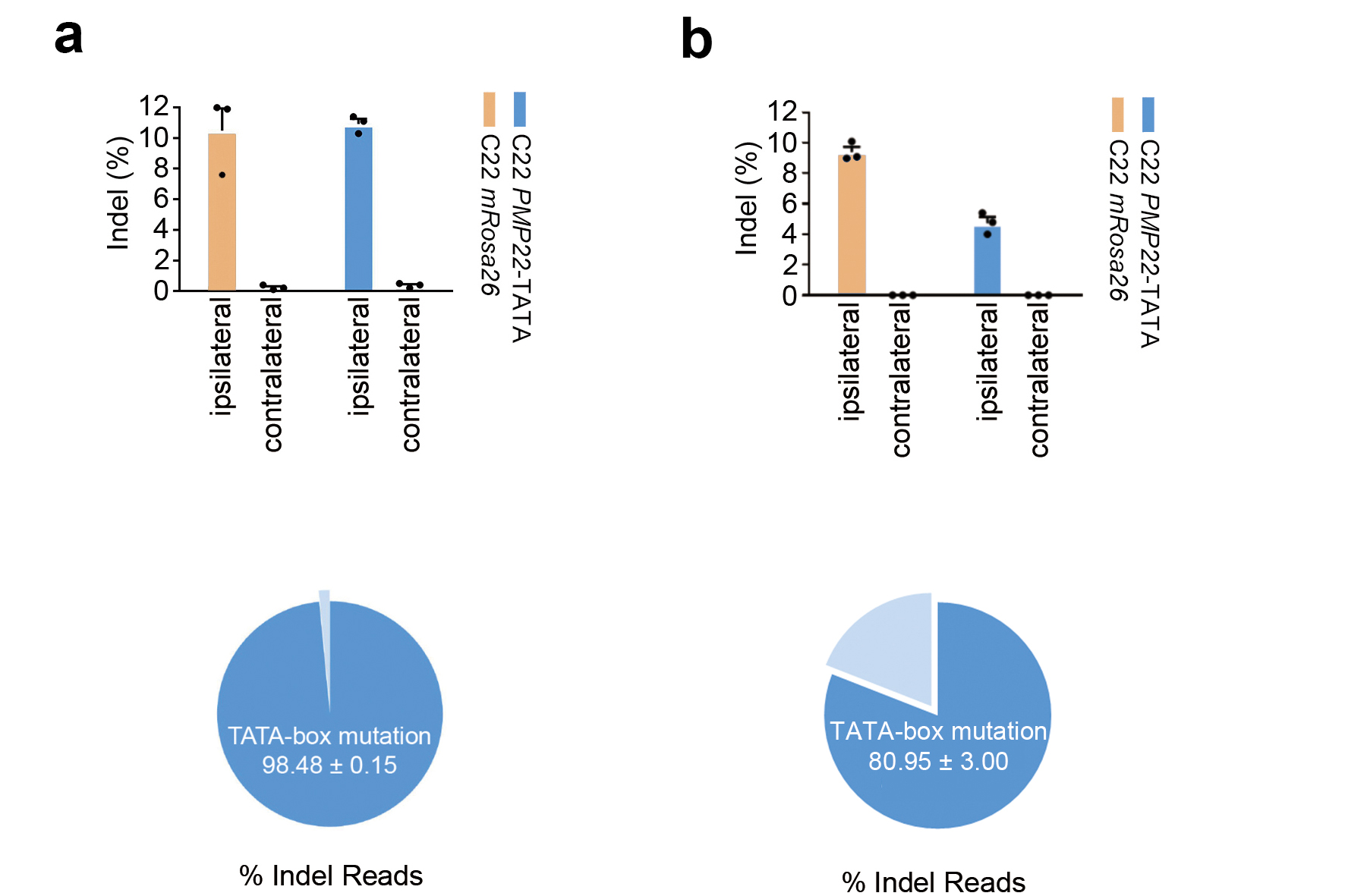


**Supplementary Fig. 4 Targeted deep sequencing of on-target analysis of PMP22-TATA or mRosa26 of RNP administered sciatic nerves of C22 mice**

Indel frequencies measured by targeted deep sequencing in sciatic nerves treated with *mRosa26* or *PMP22*-TATA RNP complexes at (a) p6 and (b) p21 (*n* = 3 for both treatments)

**
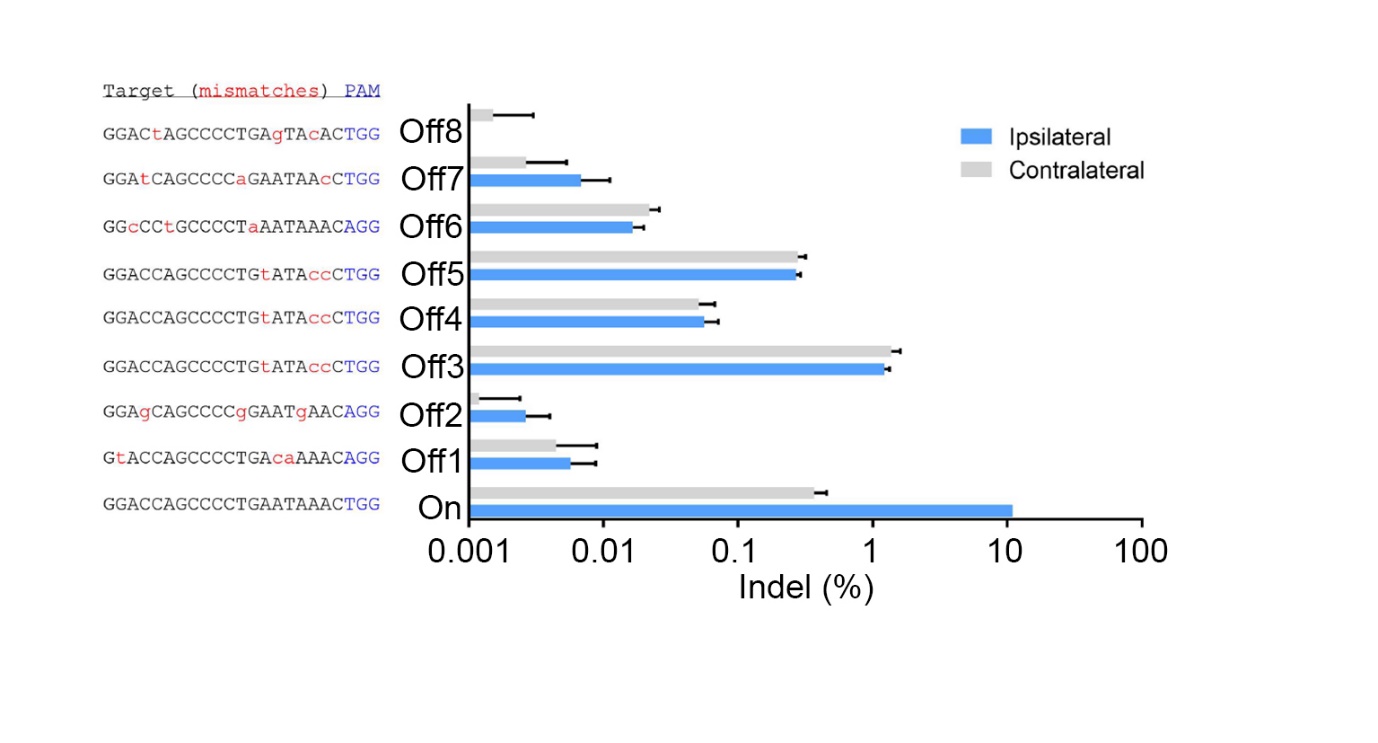
**

**Supplementary Fig. 5 *In silico* off-target analysis of *PMP22*-TATA RNP in sciatic nerves of C22 mice treated with *PMP22*-TATA RNP**

Indel frequencies in sciatic nerve, determined by targeted deep sequencing, at *PMP22*-TATA RNP on-target and off-target sites identified by *in silico* off-target analysis. The mismatched nucleotides are shown in red and PAM sequences are shown in blue.


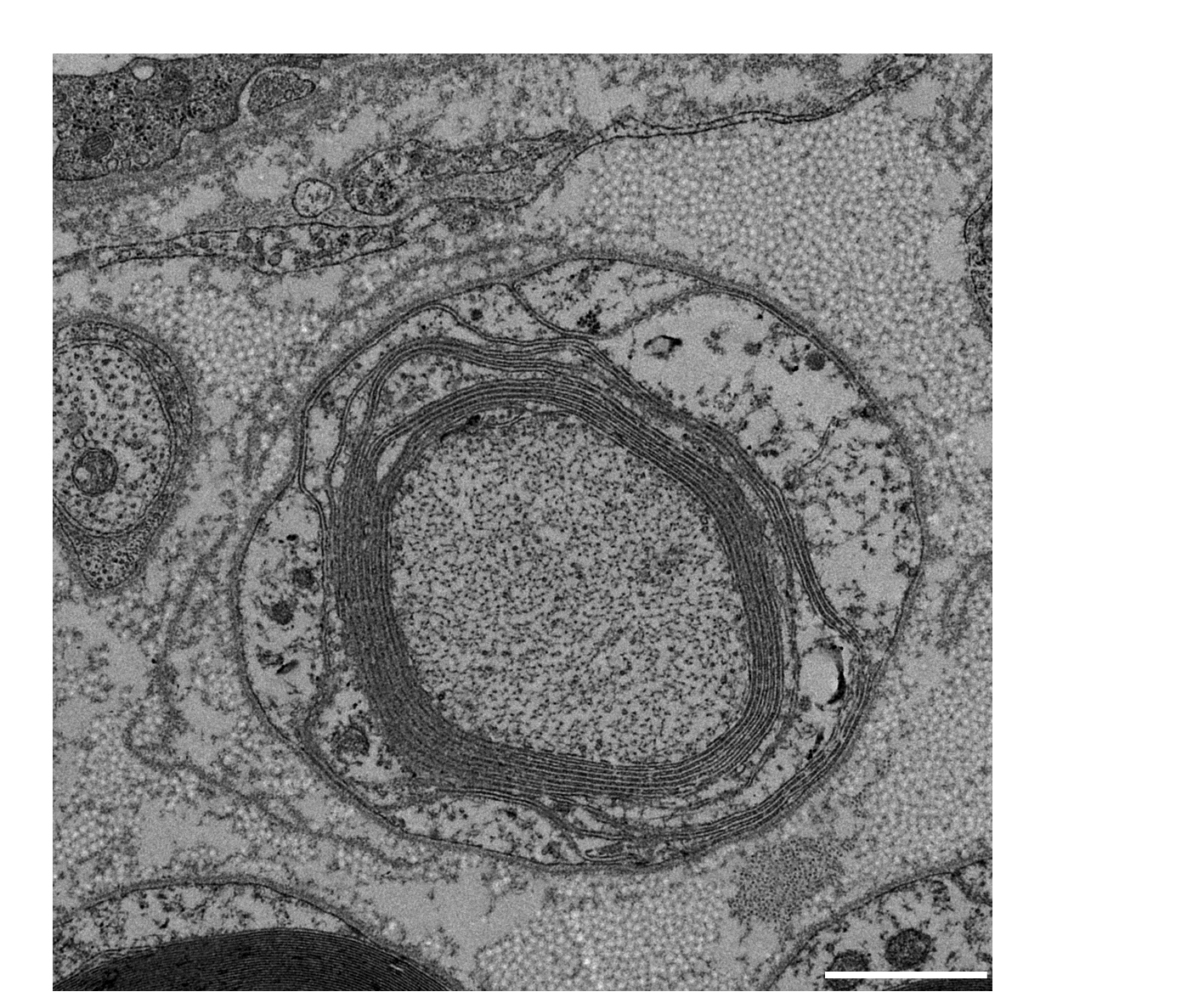


**Supplementary Fig. 6 Onion bulb in a C22 mouse**

A classical onion bulb from a C22 mouse (scale bar = 1μm).


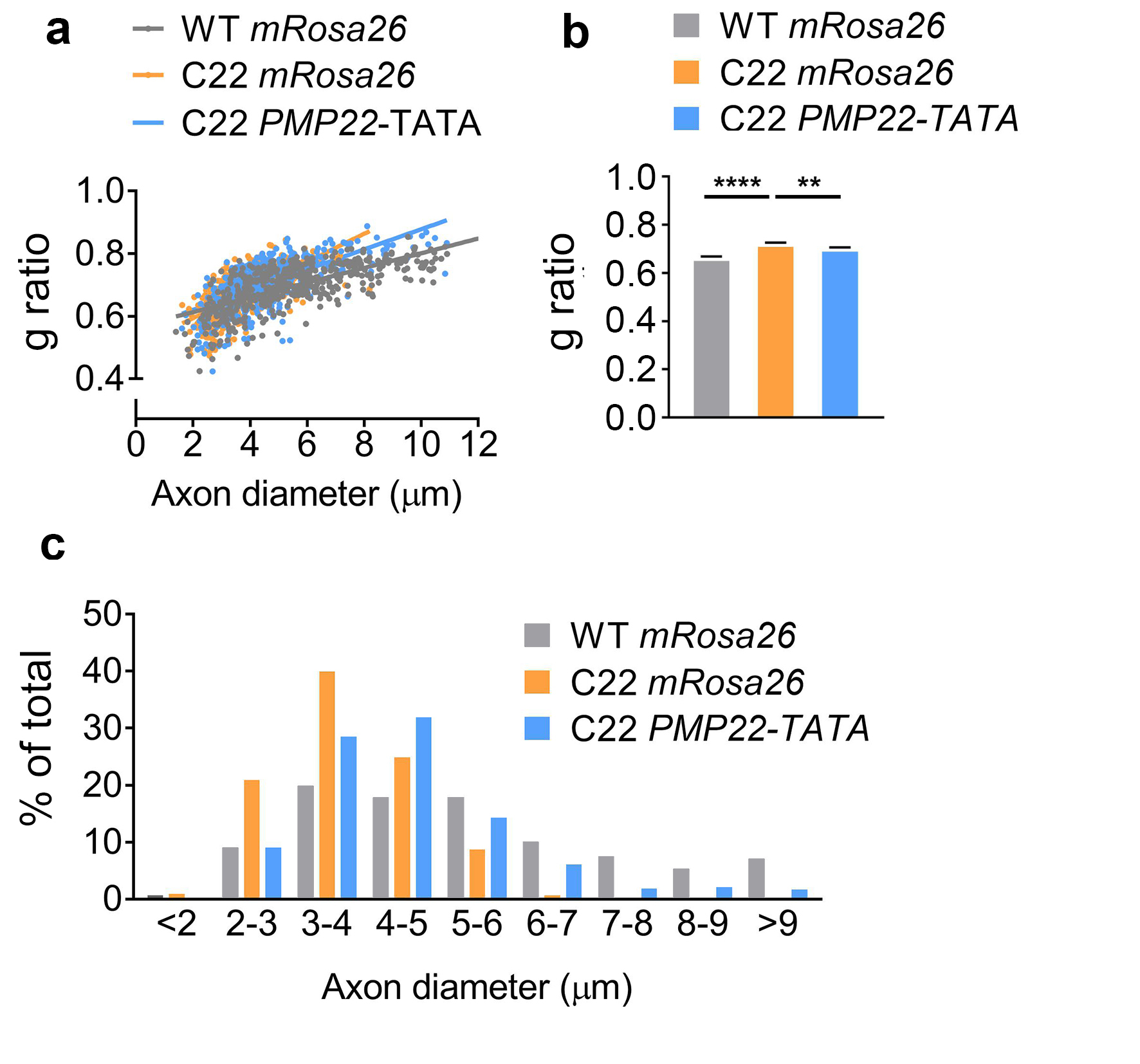


**Supplementary Fig. 7 Myelin and axon diameter restoration by *PMP22*-TATA RNP in C22 mice**

(a) A scatter plot of the *g*-ratio of *mRosa26* RNP treated WT or mRosa26 or *PMP22*-TATA RNP treated sciatic nerves of C22 mice. (b) The mean g-ratios of *mRosa26* RNP treated WT or mRosa26 or *PMP22*-TATA RNP treated sciatic nerves of C22 mice. (c) Distribution profile of myelinated axonal diameter *mRosa26* or *PMP22*-TATA RNP treated sciatic nerves of C22 mice. n=5


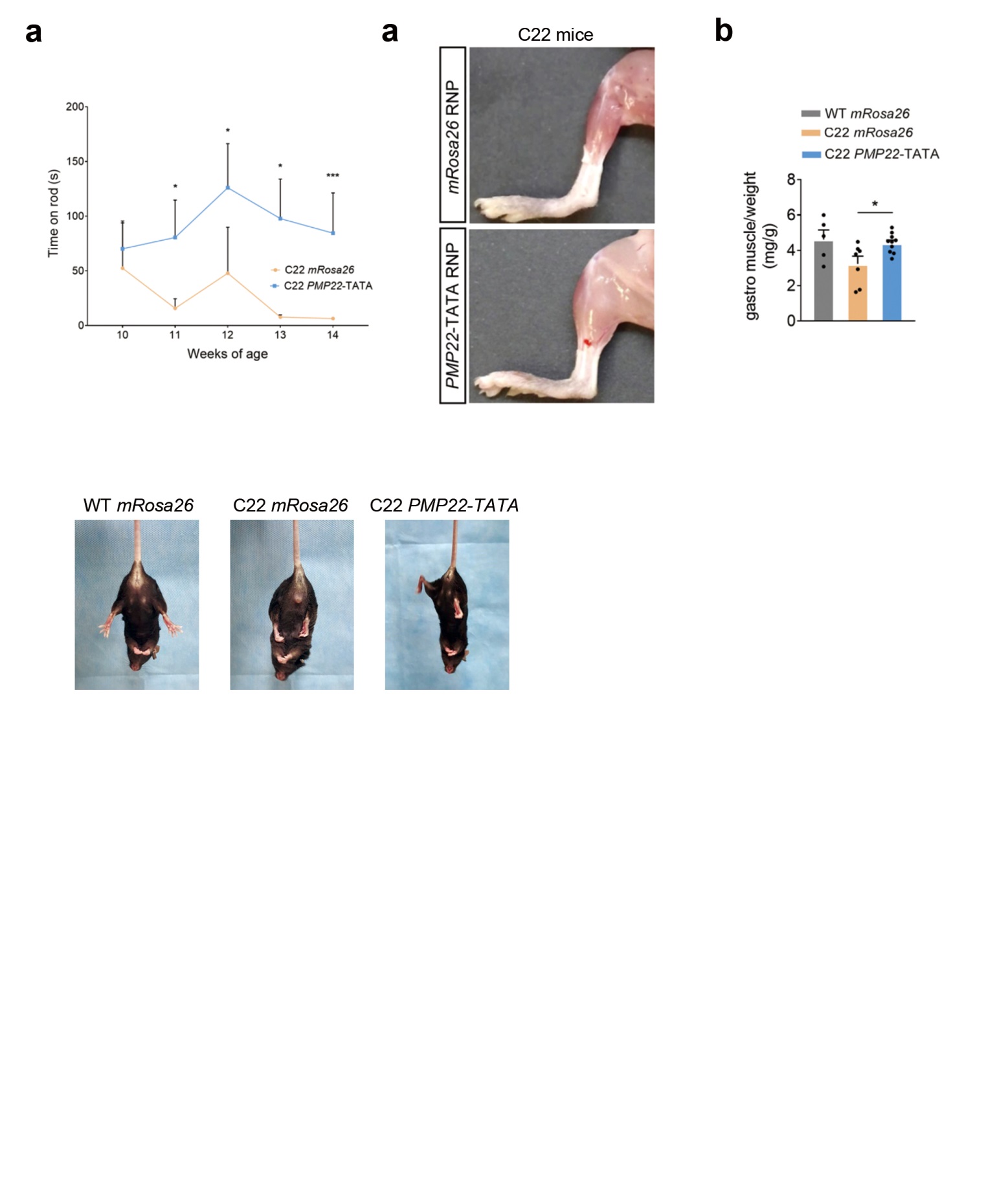


**Supplementary Fig. 8 Increased muscle mass of C22 mice by *PMP22*-TATA RNP**

(a) Representative images of hindlimb skeletal muscle of C22 mice treated with *mRosa26* or *PMP22*-TATA RNP at 15 weeks post treatments. (b) The ratio of gastrocnemius muscle weight/body weight of C22 mice at 15 weeks post treatments with *mRosa26* or *PMP22*-TATA RNP. *n*=7 for *mRosa26* RNP, *n*=10 for *PMP22*-TATA RNP. Scale bar, 20μm; *, *p*<0.05; **, *p*<0.01; ***, *p*<0.005.
